# VHL-Mediated Regulation of CHCHD4 and Mitochondrial Function

**DOI:** 10.3389/fonc.2018.00388

**Published:** 2018-10-04

**Authors:** Thomas Briston, Jenna M. Stephen, Luke W. Thomas, Cinzia Esposito, Yuen-Li Chung, Saiful E. Syafruddin, Mark Turmaine, Lucas A. Maddalena, Basma Greef, Gyorgy Szabadkai, Patrick H. Maxwell, Sakari Vanharanta, Margaret Ashcroft

**Affiliations:** ^1^Division of Medicine, Centre for Cell Signalling and Molecular Genetics, University College London, London, United Kingdom; ^2^Department of Medicine, University of Cambridge, Cambridge, United Kingdom; ^3^Cancer Research UK Cancer Imaging Centre, Institute of Cancer Research London, London, United Kingdom; ^4^Medical Research Council Cancer Unit, Hutchison/MRC Research Centre, University of Cambridge, Cambridge, United Kingdom; ^5^Division of Biosciences, Department of Cell and Developmental Biology, University College London, London, United Kingdom; ^6^The Francis Crick Institute, London, United Kingdom; ^7^Department of Biomedical Sciences, University of Padua, Padua, Italy; ^8^Cambridge Institute for Medical Research, University of Cambridge, Cambridge, United Kingdom

**Keywords:** von Hippel-Lindau protein (pVHL), hypoxia inducible factor, mitochondria, bioenergetics, metabolism, CHCHD4, respiratory chain

## Abstract

Dysregulated mitochondrial function is associated with the pathology of a wide range of diseases including renal disease and cancer. Thus, investigating regulators of mitochondrial function is of particular interest. Previous work has shown that the von Hippel-Lindau tumor suppressor protein (pVHL) regulates mitochondrial biogenesis and respiratory chain function. pVHL is best known as an E3-ubiquitin ligase for the α-subunit of the hypoxia inducible factor (HIF) family of dimeric transcription factors. In normoxia, pVHL recognizes and binds hydroxylated HIF-α (HIF-1α and HIF-2α), targeting it for ubiquitination and proteasomal degradation. In this way, HIF transcriptional activity is tightly controlled at the level of HIF-α protein stability. At least 80% of clear cell renal carcinomas exhibit inactivation of the *VHL* gene, which leads to HIF-α protein stabilization and constitutive HIF activation. Constitutive HIF activation in renal carcinoma drives tumor progression and metastasis. Reconstitution of wild-type VHL protein (pVHL) in pVHL-defective renal carcinoma cells not only suppresses HIF activation and tumor growth, but also enhances mitochondrial respiratory chain function via mechanisms that are not fully elucidated. Here, we show that pVHL regulates mitochondrial function when re-expressed in pVHL-defective 786O and RCC10 renal carcinoma cells distinct from its regulation of HIF-α. Expression of CHCHD4, a key component of the disulphide relay system (DRS) involved in mitochondrial protein import within the intermembrane space (IMS) was elevated by pVHL re-expression alongside enhanced expression of respiratory chain subunits of complex I (NDUFB10) and complex IV (mtCO-2 and COX IV). These changes correlated with increased oxygen consumption rate (OCR) and dynamic changes in glucose and glutamine metabolism. Knockdown of HIF-2α also led to increased OCR, and elevated expression of CHCHD4, NDUFB10, and COXIV in 786O cells. Expression of pVHL mutant proteins (R200W, N78S, D126N, and S183L) that constitutively stabilize HIF-α but differentially promote glycolytic metabolism, were also found to differentially promote the pVHL-mediated mitochondrial phenotype. Parallel changes in mitochondrial morphology and the mitochondrial network were observed. Our study reveals a new role for pVHL in regulating CHCHD4 and mitochondrial function in renal carcinoma cells.

## Introduction

Dysregulated mitochondrial function is associated with a broad range of diseases including renal disease (1) and cancer (2). Mitochondria are best known as the sites for cellular oxygen consumption, generating chemical energy in the form of adenosine triphosphate (ATP). In fact, mitochondria are not only central to controlling many cellular metabolic pathways, but form an intracellular network enabling them to move, interface with other organelles (nucleus, endoplasmic reticulum, lysosome) and signal to/from different cellular compartments and machinery (3, 4). Thus, mitochondria are involved in regulating a broad range of cellular activities.

Mitochondria communicate with the oxygen-sensing machinery and are involved in regulating the cellular response to hypoxia (5). The HIF (HIF-1 and HIF-2) dimeric transcription factors are central to mediating hypoxia responses in cells. Hypoxia and increased HIF activation occurs in most cancers, leading to the upregulation of a range of genes involved in diverse cellular processes including metabolism and cell survival (6, 7). HIF comprises a tightly regulated HIF-α subunit (HIF-1α and HIF-2α) and constitutively expressed HIF-1β subunit (7). Under normal oxygen levels, HIF-α protein is continuously synthesized and degraded. Targeting of HIF-α protein for degradation is achieved through hydroxylation of conserved proline residues, catalyzed by the oxygen-sensing dioxygenases, prolyl hydroxylase domain (PHD) enzymes (7–9). Recognition of the hydroxylated residues in HIF-α subunits is under the control of the von Hippel-Lindau (pVHL) tumor suppressor protein (10–12). pVHL forms the substrate recognition and catalytic component of an E3-ligase complex which functions to poly-ubiquitinate HIF-α subunits, targeting them for degradation by the 26S proteasome (8, 11, 12).

Inactivation of *VHL* occurs in a large percentage of patients with clear cell renal cell carcinomas (the most common form of kidney cancer) (13). Loss of pVHL tumor suppressor function promotes unopposed HIF-α stabilization and constitutive HIF activation which is associated with tumor progression (14). Re-constitution of wild-type pVHL or patient-derived mutant pVHL proteins into pVHL-defective renal carcinoma cells has proved a useful approach for investigating pVHL function (15–19). Interestingly, re-expression of pVHL in renal carcinoma cells increases the expression and activity of certain respiratory chain subunits including complex IV (CIV) subunits, mtCO-2 and COX IV (also known as COX4I1, COX4-1, and COX IV-1) [(18, 19), Supplementary Table 1], increases oxygen consumption rate (OCR) and mitochondrial DNA (mtDNA) content (20, 21). Knockdown of HIF-1α or HIF-2α in pVHL-deficient renal carcinoma cells has been shown to enhance basal OCR, mtDNA content and increase COX IV protein levels (20, 21). Collectively, these previous studies have led to the idea that constitutive HIF activation in the context of pVHL-defective renal carcinoma cells negatively regulates mitochondrial function (20). However, increased expression of mitochondrial respiratory chain subunits observed upon pVHL re-expression in pVHL-defective renal carcinoma cells is not HIF-α-dependent (21), suggesting that pVHL (positively) regulates mitochondrial function independently of its HIF-regulatory role through molecular mechanisms that have yet to be fully elucidated.

Previously, we discovered that the coiled-coil helix coiled-coil helix (CHCH) domain 4.1 (CHCHD4) mitochondrial import protein is crucial for regulating intracellular oxygenation, mitochondrial localization, and morphology (22, 23). CHCHD4 [also known as MIA40 (24)] provides an import and oxidoreductase-mediated protein folding function as a key component of the disulphide relay system (DRS) within the mitochondrial intermembrane space (IMS) (22–27). CHCHD4 substrates contain a twin-CX_n_C motif and include respiratory chain subunits of complex I (CI) and CIV (22, 28–30).

Here, we further explore the role of pVHL in regulating mitochondrial function, bioenergetics, and morphology. We investigate effects on CHCHD4, metabolism and the contribution of HIF-2α. We show that pVHL increases the expression of CHCHD4, respiratory chain subunits known to be CHCHD4 substrates (28, 29) and promotes changes in mitochondrial morphology when re-expressed in pVHL-defective renal carcinoma cells. Alongside, we show increased OCR and dynamic changes in glucose and glutamine utilization. Using a panel of pVHL mutants (R200W, N78S, S183L and D126N) that are unable to degrade HIF-α, but promote differential effects on glycolytic metabolism (31), we show that these mutants also differentially affected the pVHL-mediated mitochondrial phenotype. Collectively, our data provide new molecular insight into the role of pVHL in regulating mitochondrial function, bioenergetics and morphology in renal carcinoma cells.

## Results

### pVHL re-expression regulates mitochondrial protein expression and increases basal OCR

**Figure 1:**
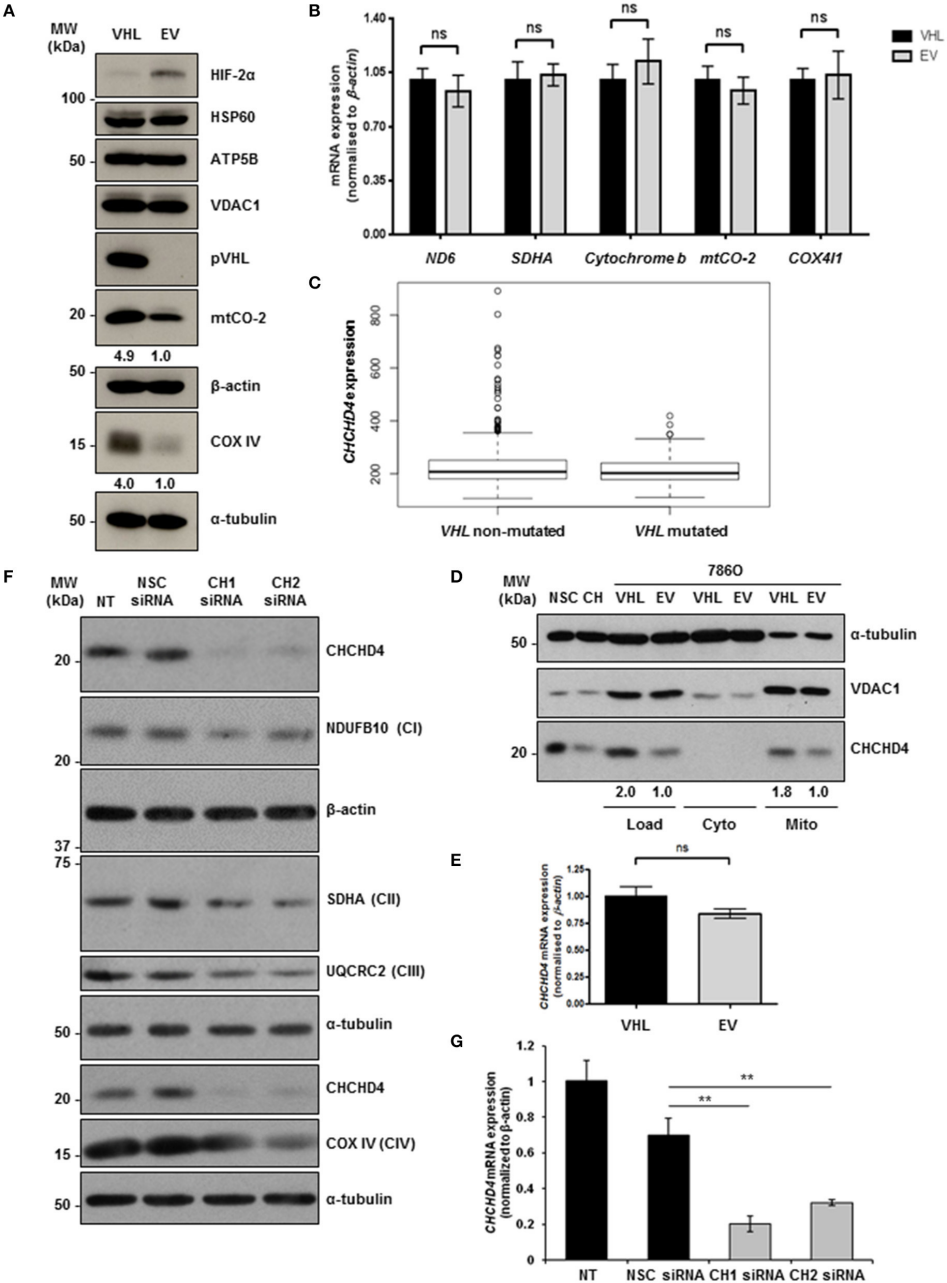
pVHL re-expression regulates mitochondrial protein expression. **(A)** Western blot analysis of mitochondrial protein expression in 786O-VHL (VHL, stably re-expressing pVHL) cells and 786O-EV (EV, vector control) cells. α-tubulin and β-actin were used as load controls. Densitometric analysis of western blots show fold change in mtCO-2 and COXIV protein levels normalized to β-actin and α-tubulin load controls respectively. **(B)** Graph shows relative expression of mRNA transcripts (*ND6, SDHA, cytochrome b, mtCO-2*, and *COX IV*) in 7860-VHL (VHL) and 786O-EV (EV) cells, measured using RT-qPCR. Data were analyzed using the comparative Ct method (*n* = 4). **(C)** Boxplot of *CHCHD4* expression in *VHL* mutated and *VHL* non-mutated ccRCC. TGCA-KIRC data was downloaded from cBioportal. *n* = 534 patients were divided into *VHL* mutant (*n* = 202) and *VHL* non-mutant (*n* = 332) and analyzed for *CHCHD4* expression in each group. Mean expression *VHL* non-mutated = 235.8, *VHL* mutated = 212.0. Median values are shown for each group (black line), vertical size of the boxes are the interquartile range (IQR), and whiskers represent 1.5 × IQR. Data were analyzed using a *t-*test with Welch's correction *p* = 0.00034. **(D)** Western blots show CHCHD4 protein expression in total (Load), cytoplasmic (Cyto), and mitochondrial (Mito) fractions from 786O-VHL (VHL) and 7860-EV (EV) cells. HCT116 cells transiently expressing non-silencing control siRNA (NSC) or CHCHD4 siRNAs (CH), were used as controls for CHCHD4 protein expression (left two lanes). α-tubulin and VDAC1 were used as cytoplasmic and mitochondrial protein markers respectively. **(E)** Graph shows relative expression of *CHCHD4* mRNA expression in 7860-VHL and 786O-EV cells, measured using RT-qPCR. Data analyzed using the comparative Ct method. Data are presented as mean ± S.E.M. *n* = 4 (not significant (n.s.) *p* > 0.05). **(F)** Western blots show expression of mitochondrial proteins CHCHD4 and respiratory chain subunits NDUFB10 (CI), SDHA (CII), UQCRC2 (CIII), and COX IV (CIV) in 786O-EV cells transiently expressing non-silencing control siRNA (NSC, 20 nM) or two independent CHCHD4 siRNAs (CH1 and CH2, 20 nM). α-tubulin and β-actin were used as a load controls. **(G)** Relative expression of *CHCHD4* mRNA in cells described in **(A)**, measured using RT-qPCR. Data were analyzed using the comparative Ct method. Data are presented as mean ± S.D. *n* = 3 (**p < 0.01).

To explore the role of pVHL in the regulation of mitochondrial function, we used matched 786O renal carcinoma cell lines stably expressing either an empty vector control (786O-EV) or re-expressing wild-type pVHL (786O-VHL) (15). 786O parental cells harbor a single nucleotide inactivating deletion in *VHL*, and as a result express constitutively stabilized HIF-2α but do not exhibit detectable HIF-1α protein (32) (Figure 1A). Consistent with Hervouet et al. (21), we found that pVHL re-expression enhanced the basal expression of respiratory chain subunits of both nuclear and mitochondrial genetic origin, including the CIV subunits mtCO-2 and COX IV (Figure 1A). The expression of a number of distinct non-electron transporting mitochondrial proteins, voltage-dependent anion channel 1 (VDAC1), heat shock protein 60 (HSP60) and ATP5B were not changed by pVHL re-expression (Figure 1A). Evaluation of a panel of both nuclear (*SDHA* and *COX4I1*) and mitochondrial encoded (*ND6, cytochrome b* and *mtCO-2*) transcripts encoding mitochondrial proteins including those major regulators of mitochondrial translation showed no significant change (Figure 1B and Supplementary Figure 1), indicating the observed increase in mitochondrial respiratory subunit expression in the 786O-VHL cells was not due to changes in mRNA.

**Figure 2:**
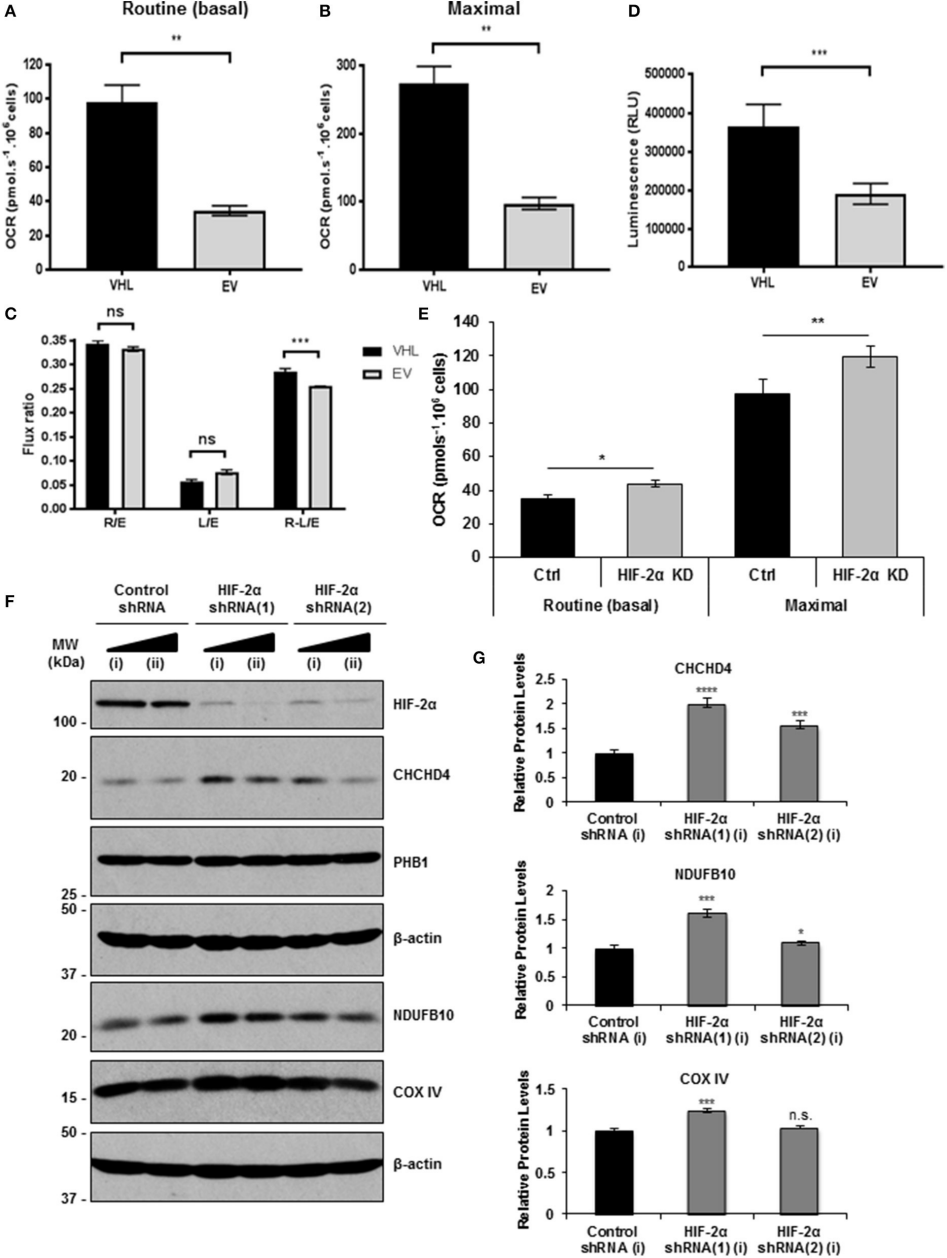
pVHL re-expression or HIF-2α knockdown increases basal OCR. **(A,B)** Graphs shows routine (basal) OCR **(A)** and maximal respiratory capacity **(B)** in 786O-VHL (VHL) and 786O-EV (EV) cells. OCR (pmol.s^−1^) was corrected for cell number (per 10^6^ cells) and non-mitochondrial respiration (*n* = 4). **(C)** Graph shows respiratory flux ratios (R/E, routine control ratio; L/E, leak control ration; R-L/E, net-routine control ratio) calculated from OCR data described in **(A)** (*n* = 4). **(D)** Graph shows luminescence (RLU) as a measure of total cellular ATP content in 786O-VHL (VHL) and 786O-EV (EV) cells, normalized to cell number (*n* = 3). **(E)** Graph show routine (basal) OCR and maximal respiratory capacity in 786O-EV cells transiently expressing a non-silencing control siRNA (Ctrl) or HIF-2α siRNA (HIF-2α KD). Data are presented as mean ± S.D. *n* = 4 (*p < 0.05) and (**p < 0.01). **(F)** Western blots show HIF-2α, COXIV and CHCHD4 protein levels in two independent 786O cell pools (i and ii) stably transduced with increasing amounts of control shRNA and two independent HIF-2α shRNAs(1) and (2). Data are representative of three independent experiments. **(G)** Graphs show relative levels of CHCHD4, NDUFB10 and COX IV protein from densitometric analysis of western data from experiments described in **(F)**. Values were normalized to the load. Data are presented as mean ± S.D. (n.s. *p* > 0.05, *p < 0.05, ***p < 0.001, and ****p < 0.0001).

CHCHD4 regulates the import of a range of mitochondrial proteins including respiratory chain subunits of CI and CIV (28–30). Bioinformatics analyses of the Cancer Genome Atlas (TCGA-KIRC) data collection showed significantly increased *CHCHD4* expression in clear cell renal cell carcinoma (ccRCC) designated as *VHL* non-mutated compared with those designated as *VHL* mutated in the collection (Figure 1C). Interestingly, we found that pVHL re-expression enhanced CHCHD4 protein levels without significantly affecting *CHCHD4* mRNA in 786O cells (Figures 1D,E). Furthermore, CHCHD4 knockdown reduced the levels of respiratory chains subunits of CI (NDUFB10), complex II (CII, SDHA), complex III (CII, UQCRC2), and CIV (COX IV) in 786O cells (Figures 1F,G). Previously we have shown that elevated CHCHD4 expression significantly increases basal OCR and ATP levels in normoxia in tumor cells expressing functional pVHL (22). Notably, we found that prolonged exposure of 786O-EV cells to hypoxia (1% O_2_, 72 h) reduced the growth of 786O-VHL cells but had no significant effect on the growth of 786O-EV cells (Supplementary Figure 2B), indicating that pVHL re-expression may lead to an increased reliance on oxygen (and oxidative phosphorylation) for cell growth. In relation to this, and consistent previous studies (20, 21), we found that pVHL re-expression significantly increased basal OCR, maximal respiratory capacity (Figures 2A–C and Supplementary Figure 2) and ATP levels (Figure 2D). Collectively, these data indicate that pVHL affects the expression of CHCHD4 as well as the expression of other CHCHD4-regulated respiratory chain subunits, basal OCR and ATP levels.

Knockdown of HIF-α in renal carcinoma cells has been shown to increase mitochondrial COX IV protein expression (21) and basal OCR (20), implying a role for constitutive HIF-α in negatively regulating mitochondrial respiration (20). In agreement, we found that HIF-2α knockdown (HIF-2α KD) significantly enhanced basal OCR and maximal respiratory capacity in 786O cells (Figure 2E). As expected, in parallel, we observed reduced expression of HIF-2α protein and *HIF2A* mRNA levels, and a reduction in the levels of the HIF-2 target, GLUT-1 (Supplementary Figures 3A,B). Furthermore, we found that stable HIF-2α knockdown led to elevated CHCHD4, NDUFB10, and COX IV protein levels (Figure 2F). These effects were more obvious with HIF-2α shRNA(1) (Figures 2F,G) which gave better knockdown than HIF-2α shRNA(2) (Supplementary Figure 3C). Alongside, we observed significantly reduced expression of *HIF2A* and HIF-2 target genes (*VEGFa* and *CCND1*) (Supplementary Figure 3D). These data suggest that constitutive HIF-2α affects the expression of CHCHD4 and mitochondrial respiratory chain subunits NDUFB10 and COX IV in (pVHL-defective) renal carcinoma cells, and this is associated with HIF-2α-dependent regulation of OCR.

### pVHL re-expression promotes dynamic changes in glucose and glutamine metabolism

**Figure 3:**
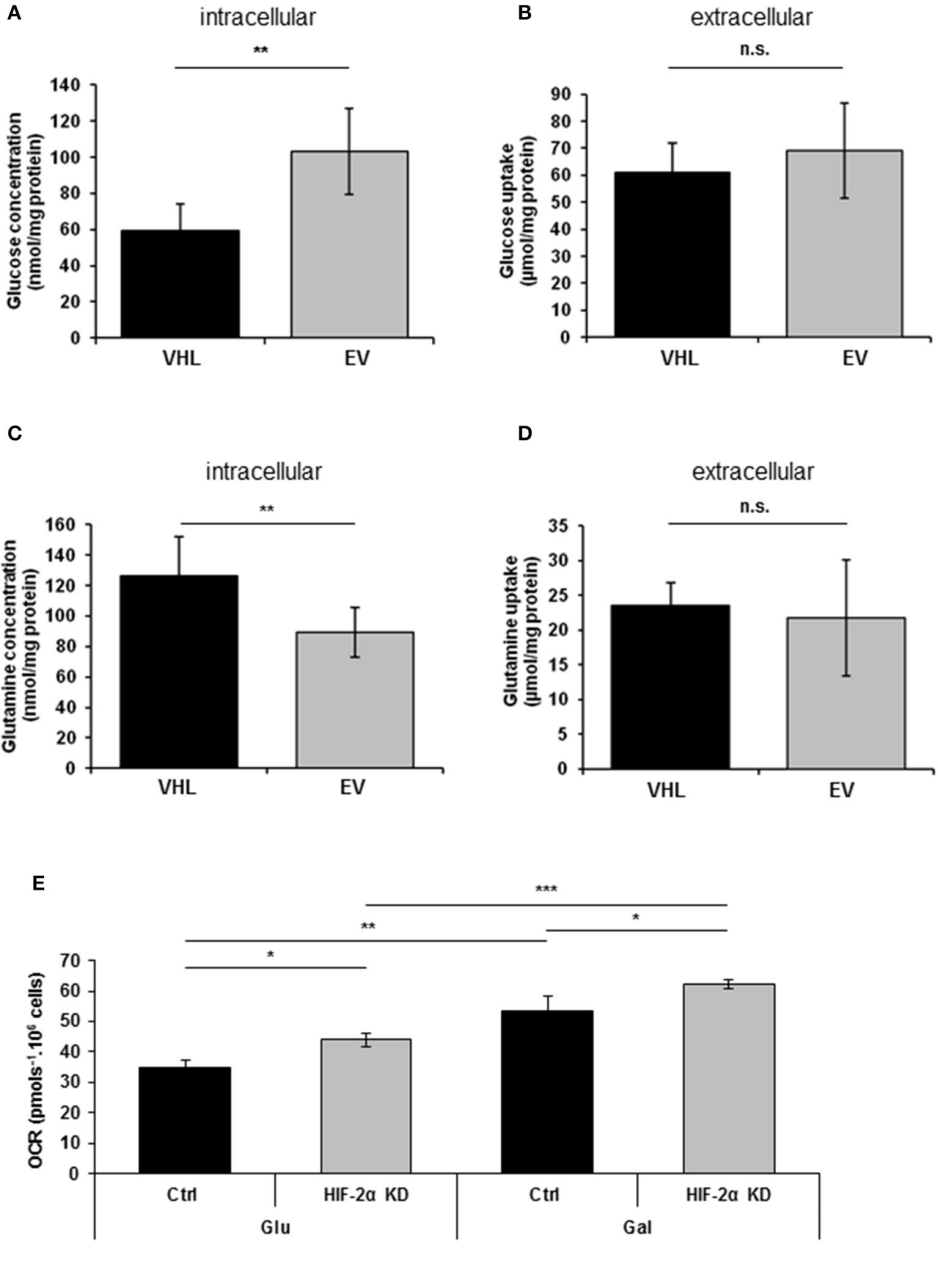
pVHL re-expression promotes dynamic changes in glucose and glutamine metabolism. **(A–D)** Graphs show, **(A)** intracellular glucose levels (*n* = 8), **(B)** glucose uptake from extracellular media (*n* = 8), **(C)** intracellular glutamine levels (*n* = 7), and **(D)** glutamine uptake from extracellular media (*n* = 7) in 786O-VHL (VHL) and 786O-EV (EV) cells measured by magnetic resonance spectroscopy (MRS). *n* = 7–8. **(E)** Graph shows basal OCR in 786O-EV cells transiently expressing a non-silencing control siRNA (Ctrl) or HIF-2α siRNA (HIF-2α KD) and cultured in glucose (Glu) or glucose–free (galactose, Gal) media. Data are presented as mean ± S.D. (n.s. *p* > 0.05, *p < 0.05, **p < 0.01, and ***p < 0.001).

Previous studies have reported that re-expressing pVHL in RCC10 renal carcinoma cells results in changes in glycolytic metabolism (31, 33). Here, we performed metabolomics analysis of 786O-EV and 786O-VHL cells. Consistent with the observed pVHL-mediated increase in respiratory chain subunit expression and enhanced OCR (Figures 1, 2), we found a significant decrease in intracellular glucose levels alongside a corresponding significant increase in intracellular glutamine levels in 786O-VHL cells compared with 786O-EV cells (Figures 3A,C). However, we found no significant change in glucose or glutamine uptake, which was measured in parallel by analyzing the media from 786O-VHL and 786O-EV cells (Figures 3B,D). Collectively, these data indicate that pVHL re-expression leads to a change in glucose and glutamine utilization consistent with increased respiratory drive.

Culturing tumor cells in glucose-free (galactose-containing) media forces them to utilize the respiratory chain and produce ATP via oxidative phosphorylation (34). Thus to evaluate the contribution of HIF-2α to respiration in the presence and absence of glucose, we measured basal OCR in 786O cells in the context of HIF-2α knockdown (Figure 3E). As anticipated, shifting cells to glucose-free (galactose-containing) media significantly increased basal OCR (Figure 3E), and was observed for both control and HIF-2α knockdown cells (Figure 3E). Notably, this effect on basal OCR was more significant for HIF-2α knockdown cells than control cells (Figure 3E). Moreover, compared to control cells for each condition, HIF-2α knockdown caused a similar increase in basal OCR in both glucose and glucose-free (galactose-containing) media, indicating that HIF-2α loss in (pVHL defective) renal carcinoma cells can promote increased mitochondrial respiration under different metabolic environments.

### pVHL mutants differentially regulate mitochondrial protein expression, mtDNA copy number and ATP levels

**Figure 4:**
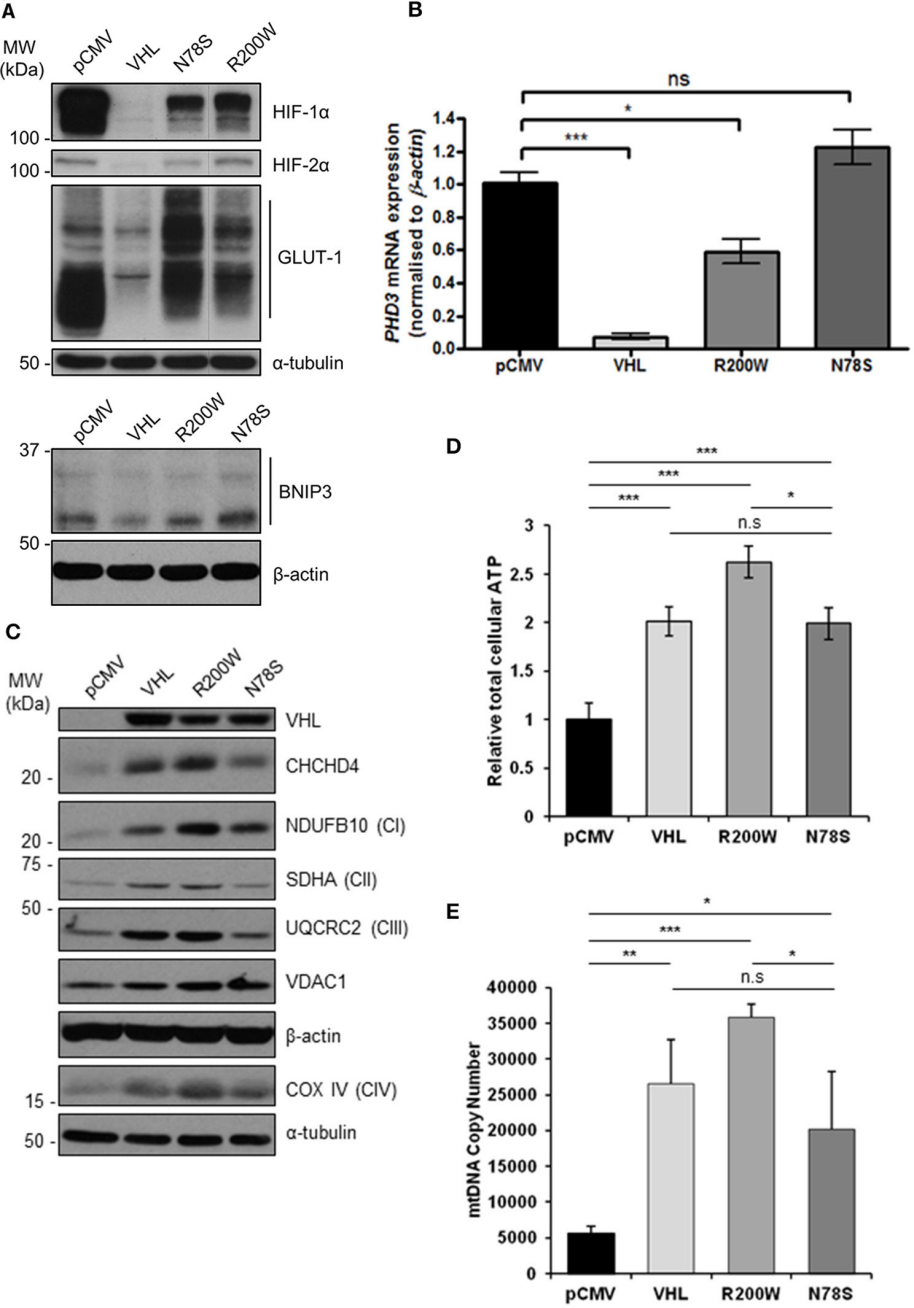
pVHL mutants differentially regulate mitochondrial protein expression, mtDNA copy number and ATP levels. **(A)** Western blots show HIF-1α, HIF-2α, GLUT-1, and BNIP3 protein levels in RCC10 cells expressing empty vector (pCMV), wild type pVHL (VHL), or pVHL mutants (R200W or N78S). α-tubulin and β-actin were used as load controls. **(B)** Relative expression of *PHD3* mRNA in RCC10 cells described in **(A)**, measured using RT-qPCR. Data were analyzed using the comparative Ct method. Data are presented as mean ± S.E.M. *n* = 3 (n.s. *p* > 0.05, *p < 0.05, and ***p < 0.001). **(C)** Western blots show expression of mitochondrial proteins CHCHD4 and VDAC1, and respiratory chain subunits NDUFB10 (CI), SDHA (CII), UQCRC2 (CIII), COX IV (CIV) in RCC10 cells described in **(A)**. pVHL expression was assessed as a control for re-expression, and β-actin and α-tubulin were used as load controls. **(D)** Graph shows total cellular ATP content in RCC10 cells expressing wild type pVHL (VHL) or pVHL mutants (R200W or N78S), normalized to cell number (*n* = 4). **(E)** Graph shows mtDNA copy number in RCC10 cells expressing pVHL variants, calculated using the ratio of expression of mitochondrial *ND1* gene to the single copy nuclear gene, β*2M* to by RT-qPCR. Data in **(D,E)** are presented as mean ± S.D. *n* = 6 (n.s. *p* > 0.05, *p < 0.05, **p < 0.01, and ***p < 0.001).

To confirm the pVHL-dependent mitochondrial effects we observed in 786O cells (Figures 1–3 and Supplementary Figures 1–3), we used RCC10 cells (35). RCC10 cells harbor an inactivating deletion in *VHL* (35), but in contrast to the 786O cells, RCC10 cells constitutively express both HIF-1α and HIF-2α proteins (Figure 4A). Pooled RCC10 cell lines independently stably expressing either control vector (pCMV), wild type pVHL (VHL), or a panel of mutant forms of pVHL proteins (R200W, N78S, D126N, and S183L) which are impaired in their ability to degrade HIF-α protein (31, 36) (Figure 4A and Supplementary Figure 4A) were used. The R200W mutation in *VHL* is associated with congenital polycythemia (37), while its tumor suppressor activities are considered to be close to wild type VHL (38). The N78S, D126N, and S183L mutations in pVHL reduce its stability, and promote differential effects on glycolytic metabolism (31). As anticipated, these pVHL mutants were defective in blocking HIF activity and unable to rescue constitutive expression of HIF targets (GLUT1, BNIP3, and *PHD3*) (Figures 4A,B). However, whether these pVHL mutations differentially regulate mitochondrial protein expression and/or function is not known.

Consistent with our observations with 786O cells (Figure 1), re-expression of pVHL in RCC10 cells led to an increase in CHCHD4, and respiratory chain subunits NDUFB10 (CI), SDHA (CII), UQCRC2 (CII), mtCO-2 (CIV), and COX IV (CIV) protein expression (Figure 4C and Supplementary Figures 4B,C), while there was no change in other mitochondrial proteins, such as VDAC1 and prohibitin-1 (PHB1) (Supplementary Figures 4B,C). Intriguingly, expression of pVHL mutants had differential effects. Analysis of the R200W mutant revealed a similar phenotype to re-expression of pVHL (Figure 4C and Supplementary Figure 4). However, compared to the R200W mutant, the N78S mutant was less able to promote an increase in the panel of mitochondrial proteins evaluated (Figure 4C and Supplementary Figure 4), total cellular ATP (Figure 4D), or mtDNA copy number (Figure 4E). Expression of the N78S mutant in RCC10 cells has previously been shown to exhibit markedly reduced glucose utilization and lactate production compared to re-expression of wild type pVHL, while two other mutants of pVHL, D126N and S183L were shown to have intermediate effects which were more similar to wild type pVHL (31). Like wild type pVHL, the D126N and S183L mutants also promoted a significant increase in mitochondrial proteins while the effect of the N78S mutant was less significant (Supplementary Figure 4). Together these data indicate the possibility that the pVHL-induced increase in mitochondrial proteins, ATP and mtDNA copy number is mechanistically separate from pVHL-mediated regulation HIF-α.

### pVHL mutants differentially regulate mitochondrial morphology and the mitochondrial network

**Figure 5:**
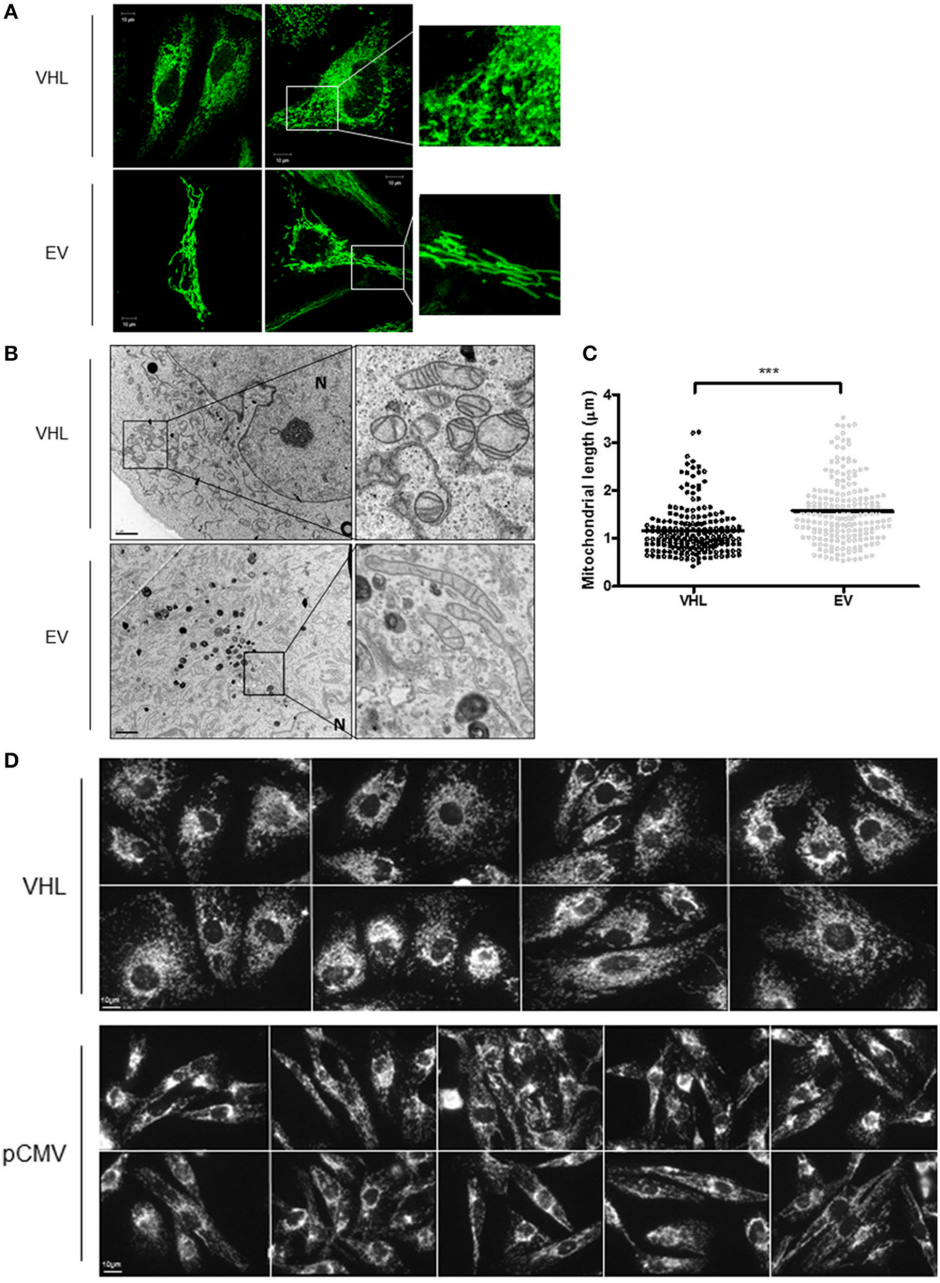
pVHL re-expression affects mitochondrial morphology and the mitochondrial network. **(A)** Confocal microscope images of 786O-EV and 786O-VHL cells stained with MitoView Green (100 nM). Images were acquired with a Zeiss 510 META confocal laser scanning microscope using a 63× oil objective. Representative images and higher magnified images (right panel) are shown. **(B)** EM images of fixed 786O-VHL and 786O-EV cells. N = nucleus. Representative images and higher magnified images (right panel) are shown. **(C)** Graph shows mitochondrial length measured from the EM images of fixed 786O-VHL (VHL) and 786O-EV (EV) cells described in **(B)**. Mitochondrial length was measured from the most highly curved membrane through the central axis to the opposite periphery. Measurements were calculated using Image J software (National Institute of Health, Maryland) after calibration of scale using the scale bar. Over 200 mitochondria were scored from independent images of 10 cells (***p < 0.001). **(D)** Confocal microscope images of RCC10 cells empty vector (pCMV) and wild type pVHL (VHL) cells. Cells were fixed and mitochondria were stained for ATP5B. Images were acquired with a Zeiss 510 META confocal laser scanning microscope using a 63× oil objective.

To further explore the relationship between the pVHL and mitochondria, 786O-VHL and 786O-EV cells were imaged using confocal and electron microscopy (EM) to assess mitochondrial morphology (Figures 5A,B). Clear differences in mitochondrial morphology between the 786O-VHL and 786O-EV cells were observed (Figures 5A,B). 786O-EV cells exhibited a more fused and elongated mitochondrial phenotype compared to 786O-VHL which appeared to have smaller and more fragmented mitochondria (Figures 5A,B). Mitochondria appeared equally dispersed throughout the cell in both 786O-EV and 786O-VHL cells (Figure 5A). In agreement with the our images obtained by live-cell confocal imaging (Figure 5A), ultrastructural analysis by EM revealed that mitochondria appeared smaller and more rounded in 786O-VHL cells, while mitochondria in 786O-EV cells appeared more elongated, thinner and more tubular (Figure 5B). Quantification of mitochondrial length from the EM images showed the average mitochondrial length was significantly smaller in 786O-VHL cells (1.15 μm) compared to 786O-EV cells (1.54 μm) (Figure 5C). Similar mitochondrial morphology changes were observed in RCC10 cells stably expressing vector control (pCMV) and wild type VHL (Figure 5D).

**Figure 6:**
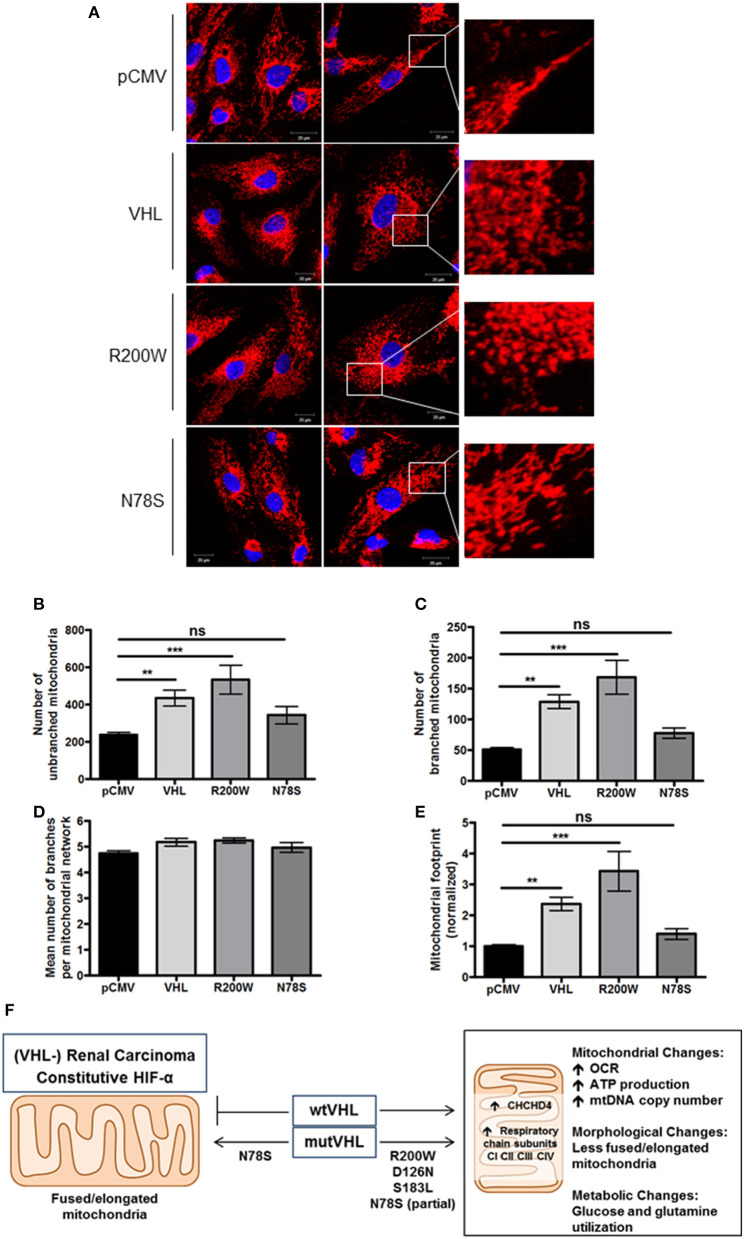
pVHL mutants differentially regulate mitochondrial morphology and the mitochondrial network. **(A)** Confocal microscope images of RCC10 cells empty vector (pCMV), wild type pVHL (VHL), or pVHL mutants (R200W or N78S). Cells were fixed and mitochondria were stained for ATP5B (red). Nuclei were stained with DAPI (blue) and images were acquired with a Zeiss 510 META confocal laser scanning microscope using a 63× oil objective. Right panels show mitochondria (red) in higher magnified images. **(B–E)** Graphs show analysis of mitochondria from ATP5B stained cells described in **(A)** using MiNA (39). Graphs show number of mitochondria that are unbranched **(B)** and branched **(C)**, mean number of branched mitochondria per network **(D)** and the mitochondrial footprint normalized to pCMV control **(E)** for each cell line as indicated. *n* = 28 (pCMV, VHL), *n* = 13 (R200W), *n* = 14 (N78S). Data are presented as mean ± S.E.M. and analyzed using a one-way ANOVA with Tukey's *post-hoc* comparison. (n.s. *p* > 0.05, **p < 0.01, and ***p < 0.001). **(F)** pVHL-mediated regulation of CHCHD4 and mitochondrial function. pVHL re-expression in VHL-defective renal carcinoma cells leads to increased expression of CHCHD4 and respiratory chain subunits (from CI to CIV), increased respiration (OCR), ATP production, mtDNA copy number, changes in metabolism (glucose and glutamine), and changes in mitochondrial morphology (less fused/elongated). pVHL mutants exhibit differential effects.

To further evaluate mitochondrial morphology, a panel of RCC10 cells stably expressing vector control (pCMV), wild type VHL (VHL), VHL mutants (R200W and N78S) described in Figure 5 were fixed, and the mitochondrial network examined using indirect immunofluorescence toward the ATP5B subunit of the F_1_F_O_-ATPase. ATP5B protein expression was unaffected by pVHL status (Figure 1A). Confocal imaging confirmed a similar change in mitochondrial morphology to 786O cells upon re-expression of pVHL (Figures 5A,D, **6A** and Supplementary Figure 5A). Images were analyzed using the mitochondrial network analysis (MiNA) macro tool (39) (Figures 6B–E and Supplementary Figure 5A). RCC10 cells expressing the R200W mutant exhibited a similar mitochondrial morphology to cells re-expressing wild type pVHL (Figure 6), as they appeared fragmented and less tubular compared to the control (pCMV) cells (Figure 6A and Supplementary Figure 5A). We observed a larger mitochondrial footprint in wild type pVHL and R200W mutant cells compared to pCMV control cells as there was significantly more area covered by mitochondrial structures (Figure 6E). In contrast, RCC10 cells expressing the N78S mutant exhibited a mitochondrial morphology similar to the control (pCMV) cells (Figure 6), as they displayed a more elongated mitochondrial appearance compared to those re-expressing wild type pVHL (Figure 6). A similar trend in results were obtained using live cell imaging of the panel of RCC10 cells stained with the mitochondrial dyes MitoTracker red (Supplementary Figures 5B–E) and MitoView green (Supplementary Figure 5F), however the mitochondrial network analysis was not statistically significant (Supplementary Figure 5). Finally, consistent with a previous study (18), and other settings (40), we found no change in the expression of major regulators of mitochondrial biogenesis including *PGC-1*α, *PGC-1*β, *NRF-1, NRF-2* (Supplementary Figures 6A–D), while mtDNA copy number (Figure 4E and Supplementary Figure 6E) and *TFAM* expression (Supplementary Figures 6F,G) was increased upon pVHL re-expression. Taken together, these data indicate that pVHL regulated mitochondrial morphology correlates with changes in mitochondrial protein expression.

## Discussion

It is clear from previous studies that pVHL regulates mitochondrial function in renal carcinoma (see Supplementary Table 1). The negative regulation of the HIF-α subunits by pVHL has understandably been the focus of most attention and study, however other HIF-independent functions of pVHL exist (41). Here, we show that pVHL regulates mitochondrial protein expression, mtDNA copy number, respiration and mitochondrial morphology (Figure 6F).

Most strikingly, we found that expression of the mitochondrial DRS protein CHCHD4 (22, 23, 29) was affected by pVHL status. CHCHD4 controls the import of a range of mitochondrial proteins into the IMS (28), and as an oxidoreductase and key component of the DRS, CHCHD4 along with ERV1/GFER introduces disulphide bonds into imported substrates (29). Previously, we have shown that elevated CHCHD4 expression increases basal OCR, controls intracellular oxygenation and regulates the mitochondrial network (22, 23). Here, pVHL re-expression led to increased levels of a range of respiratory chain subunits from CI-CIV including known CHCHD4 substrates [e.g., the CI subunit, NDUFB10 (30)]. Thus, pVHL affects CHCHD4 and mitochondrial function.

Reprogramming of glucose and glutamine metabolism occurs in renal carcinoma (42). Loss of pVHL function causes HIF-mediated enhanced glycolysis and inhibition of mitochondrial function in renal carcinoma cells (20, 43). We found that knockdown of HIF-2α in 786O cells caused elevated CHCHD4 and respiratory chain subunit expression and led to increased basal OCR, suggesting the possibility that HIF-dependent inhibition of mitochondrial function in pVHL mutated renal carcinoma involves CHCHD4. Small molecule inhibitors that target HIF show promise therapeutically in renal carcinoma (17, 44–46). Tumor cells rely on glycolysis and mitochondrial oxidative phosphorylation to survive (47). Thus, mitochondrial metabolism has become an increasingly attractive area for investigation and therapeutic exploitation in cancer (48). Our study suggests that blocking HIF-2α in (pVHL-defective) renal carcinoma induces CHCHD4 and promotes a metabolic profile that could be exploited therapeutically. Thus, investigating the importance of *CHCHD4* expression levels in renal carcinoma progression in the context of HIF dysregulation will be of particular interest. In fact our bioinformatics analysis using the Cancer Genome Atlas data collection (TCGA-KIRC) revealed that CHCHD4 expression was significantly lower in ccRCC patients designated with mutant *VHL* compared to those ccRCC patients designated with wild-type *VHL* (Figure 1C).

Along with changes in CHCHD4 and respiratory chain subunit expression, we found that pVHL re-expression caused a marked shift in fuel utilization: increased glucose and decreased glutamine utilization, consistent with the enhanced mitochondrial respiration. Using a panel of pVHL mutants N78S, D126N, and S183L that exhibit reduced stability and dysregulation of HIF-α but promote differential effects on glycolysis (31), we discovered that the pVHL-dependent effects on CHCHD4, mitochondrial protein expression and morphology directly correlated with pVHL-dependent regulation of metabolism. Our data suggest that pVHL can promote effects on CHCHD4 and mitochondrial function that are separable from its role in regulating HIF-α. Our previous study suggests that CHCHD4 controls intracellular oxygenation and the mitochondrial function upstream of HIF in the context of hypoxia (23). Future work determining the contribution of CHCHD4 and the DRS to pVHL-mediated regulation of mitochondrial protein expression, respiration, and mitochondrial morphology in the presence and absence of HIF-α will provide further mechanistic insight into the role of CHCHD4 in controlling mitochondrial function and metabolism.

## Materials and methods

### Antibodies and reagents

All chemicals were purchased from Sigma Aldrich unless otherwise specified. The following antibodies were used for immunoblotting: anti-pVHL [BD Pharmagen (Ig32; 556347; 1:1,000)], anti-α-tubulin (Sigma Aldrich; T6199; 1:5,000), anti-CHCHD4 (Sigma Aldrich; HPA034688; 1:1,000), anti-HIF-2α (Abcam; Ab199; 1:500), anti-ATP5B (Abcam; ab14730; 1:1,000), anti-mtCO-2 (Abcam; ab110258; 1:1,000), anti-β-actin (Abcam; 6276; 1:5,000), anti-HSP60 [Cell Signaling Technology (CST); #4870; 1:1,000], anti-VDAC1 (Abcam; ab15895;:1,000), anti-COX IV (Abcam; ab14744; 1:1,000), anti-PHB1 (CST; #2426; 1:1,000), anti-anti-GLUT1 (Alpha Diagnostics; GT12-A; 1:1,000), anti-DRP-1 (CST; #5391S; 1:1,000), anti-pDRP-1 (Ser^637^) (CST; #4867S; 1:1,000), anti-HIF-1α (BD Biosciences; 610959; 1:1,000). Horseradish peroxidase (HRP)-conjugated secondary antibodies were purchased from Amersham and used at 1:5,000 dilution. For immunocytochemistry anti-ATP5B (Abcam; ab14730) was used at 1:500 dilution and Alexa Fluor 568 goat anti-mouse IgG (Invitrogen; A-11031) was used at 1:5,000 dilution.

### Cell culture, siRNA transfection, and shRNA lentiviral transduction

The pVHL positive and negative 786O cells were kindly gifted to us by William Kaelin Jr (Dana-Farber Cancer Institute, Harvard Medical School), and have been described previously (15). Stable pools of RCC10 cells expressing wild-type and mutant VHL were supplemented with 0.5 μM G418 (Sigma Aldrich), and have been described previously (31). For shRNA lentivirus production, HEK293T cells were used. All cells were maintained in Dulbecco Modified Eagle Medium (DMEM; Invitrogen), supplemented with 10% FCS, L-glutamine (2 mM), penicillin (100 U/ml) and streptomycin (100 μg/ml) at standard cell culture conditions (37°C, 5% CO_2_ and 95% relative humidity). Transient transfection was performed using a non-silencing control siRNA duplex (Qiagen; 5′-AATTCTCCGAACGTGTCACGT-3′) and custom made siRNA to human HIF-2α (Dharmacon; 5′-CCCGGATAGACTTATTGCCAA-3′) (17, 49). Transient transfection with siRNA duplexes (20 nM) were performed using HiPerfect™ (Qiagen) or Lipofectamine 2000 (Invitrogen) transfection reagent according to manufacturer's instructions. Knockdown was confirmed by western blot analysis and real-time quantitative polymerase chain reaction (RT-qPCR) following 24 h siRNA incubation. For stable shRNA targeting of *HIF-2A*, we used the shRNA expression vector, sGEP [a gift from J. Zuber, IMP, Austria (50)]. The two independent *HIF-2A* shRNA oligonucleotide sequences used for cloning are listed in Supplementary Table 3, and were obtained from Sigma Aldrich. For shRNA lentivirus production, HEK293T cells were transfected with a mixture of the HIF-2A shRNA sGEP vector, psPAX2 and pMD2.G using FuGENE 6 transfection reagent (Promega E269A). The media containing the lentivirus was collected 72 h post-transfection and filtered through a 0.45 μM PVDG sterile filter. 786O cells obtained from J. Massague (MSKCC, New York) were transduced with the lentiviral supernatant in the presence of 8 μg/mL polybrene (Milipore) and selected with 4 μg/mL puromycin (Invivogen) for 48 h post-transduction. Stable selected cell pools were expanded and analyzed.

### RNA isolation and complementary DNA synthesis

Total RNA was extracted from cells using RNeasy mini kit with DNase digestion (Qiagen) as manufacturer's instructions. One microgram of total RNA was used for first strand synthesis using the qScript First-Strand cDNA Synthesis System (Quanta Biosciences). Complementary DNA (cDNA) was synthesized using the G-Storm thermocycler as per manufacturer's instructions.

### Real-time quantitative polymerase chain reaction (RT-qPCR)

Forward and reverse primers were used at a final concentration of 0.5 μM and Mesa Blue SYBR green mix (Eurogentec) was used as per manufacturer instruction. Data was analyzed using the comparative cycle threshold (Ct) method using β-actin as the internal control. Values from technical replicates were averaged and analyzed using the equation below and as published (51):

Relative expression (2 ^−ΔΔCt^) = [(Ct gene of interest – Ct internal control) sample A – (Ct gene of interest – Ct internal control) sample B].

PCR primers were obtained from Sigma Aldrich and sequences are outlined in Supplementary Table 2.

### Mitochondrial fractionation

Mitochondria were isolated using differential centrifugation. Briefly, cells from confluent 15 cm plates were trypsinised and washed in PBS. Cells were re-suspended in homogenization buffer (250 mM mannitol, 0.5 mM 2-[4-(2-hydroxyethyl)piperazin-1-yl]ethanesulfonic acid (HEPES) pH 7.4 and 0.5 mM ethylene glycol-bis(2-aminoethylether)-*N*,*N*,*N*′,*N*′-tetraacetic acid (EGTA), 100x EDTA-free protease inhibitor cocktail). Cells were homogenized with a glass-on-glass Dounce homogenizer using 100 strokes of a tight fitting pestle. A small volume of the total cell homogenate was removed and stored (load fraction). The homogenate was centrifuged at 900 *g* for 10 min to pellet nuclei and intact cells, the supernatant containing mitochondria was removed and this step was repeated and supernatant again removed. Mitochondria were sedimented following centrifugation at 9,000 g for 10 min. The supernatant was removed and stored (cytosolic fraction). The crude mitochondrial pellet was washed in homogenization buffer and centrifuged at 9,000 g for 10 min. The pellet was lysed in 1% NP40 lysis (mitochondrial fraction). All steps were performed on ice and centrifugation was performed at 4°C.

### Measurement of total cellular ATP

Cells were counted and re-suspended in complete media. To determine ATP concentration Cell-Titer Glo reagent (Promega) was used as outlined in the manufacturer's instructions. Luminescence was quantified using a Tropix TR717 microplate reader.

### High-resolution respirometry

Cellular oxygen consumption rates were measured using the Oxygraph 2K (O2K; Oroboros Instruments). Cells were trypsinised and re-suspended in HEPES-buffered DMEM and counted. Cells were added to each chamber and incubated at 37°C under constant stirring (750 rpm). Cells were allowed to reach routine baseline respiration before the addition of the first compound. Two μg/ml oligomycin, 0.5 μM increments FCCP, 0.5 μM rotenone, and 2.5 μM antimycin A were injected in sequence and the rates of oxygen consumption assessed.

### Determination of mtDNA copy number

Total DNA was isolated from cell pellets using QIAamp DNA blood mini kit (Qiagen). Abundance of mitochondrial DNA relative to nuclear genomic DNA was determined by qRT-PCR using primers for *ND1* (mitochondrial, forward 5′-TCCTCTCCCGCTCTGCACCC-3′ and reverse 5′-GGCGGGCCACCAAGGAGAAC-3′) and *beta-2-microglobulin* (β*2M*; nuclear, forward 5′- GCCCCAACGTTGTAGGCCCC-3′ and reverse 5′- AGCTAAGGTCGGGGCGGTGA-3′). Estimation of the number of copies of the mitochondrial genome relative to nuclear genome was based on the threshold cycle (Ct) as described previously (52).

### Immunostaining

Cells were plated on glass coverslips (ø 13 mm, VWR) and incubated overnight. Cells were washed twice with PBS and fixed using 4% paraformaldehyde in PBS for 15 min. Cells were then washed twice with PBS and permeabilized for 10 min using 0.5% Triton X in PBS before being blocked with IFF (1% BSA, 1% FCS in PBS) for 1 h. Coverslips were incubated overnight with primary antibody at 4°C before being washed and incubated with fluorophore conjugated secondary antibody for 1 h. Coverslips were again washed twice with PBS and incubated in 1 μg/ml DAPI solution (Biotium) for 10 min, washed and mounted using Fluoromount G (Southern Biotech). DAPI was excited at 405 nm and emitted fluorescence (peak 465 nm) collected using 435–485 band pass filter and red fluorescence captured following excitation at 543 nm and collected using a 560 nm long pass filter. Images were acquired with a Zeiss 510 META confocal laser scanning microscope using a 63× oil immersion objective.

### Confocal live-cell imaging

Cells were seeded on to glass coverslips (ø 22 mm, VWR) and Mitoview green (Biotium) used for mitochondrial imaging at a concentration of 100 nM. Mitoview green was excited at 488 nm and emitted fluorescence captured using a 505–530 band pass filter. Images were acquired with a Zeiss 510 META confocal laser scanning microscope using a 63× oil immersion objective.

### Analysis of mitochondrial network morphology

Fluorescently-labeled mitochondrial networks within cultured cells were analyzed using the Mitochondrial Network Analysis (MiNA) macro tool (39) in ImageJ. Pre-processing to enhance image quality was uniformly performed across images using the built-in contrast limited adaptive equalization, median filtering, and top-hat filtering options. Regions of interest containing single cells were selected for analysis and a mitochondrial footprint for each cell per image was generated. The mitochondrial footprint is the total area occupied by mitochondrial signal (i.e., mitochondrial structures) separated from the background, and is calculated by multiplying the number of pixels containing signal in the binarized image (only mitochondrial signal included) by the area of a pixel.

### Electron microscopy

Cells were seeded on to glass coverslips (ø 13 mm, VWR), and fixed with 2% paraformaldehyde and 1.5% glutaraldehyde in 0.1 M cacodylate buffer. Samples were post fixed with 1% osmium tetraoxide and 1.5% potassium ferrocyanine in 0.1 M cacodylate buffer. Samples were dehydrated in a graded ethanol-water series, cleared in propylene oxide and infiltrated with agar-100 resin. Ultra-thin sections were cut and collected on 300-mesh grids and stained with lead citrate. Samples were viewed in a Joel 1010 transition electron microscope and images were acquired using a Gatan Orius CDD camera. Mitochondrial length was analyzed using ImageJ measure feature, calibrated using the scale bar of each image. Analysis of up to 15–20 mitochondria from 10 cells per condition was performed. Statistical significance was calculated using an unpaired two-tailed *t-*test.

### Metabolomics analyses to determine the levels of intracellular glucose and glutamine and their uptake from the culture media

For the intracellular glucose and glutamine measurements, cells were methanol fixed, and harvested by scraping, then phase separated by the serial addition of chloroform and water, followed by centrifugation. Divalent ions were removed by the addition then removal by centrifugation of Chelex 100. Cell extracts were prepared for ^1^H-MRS by freeze drying, followed by resuspension in D_2_O and 0.75% TSP (in D_2_O) at a 14:1 ratio. For the glucose and glutamine uptake measurements, 1H-MRS were performed on 500 μl of culture media samples (starting media that has not been incubated with the cells and media samples that has been collected just before cell harvest for extractions) with the addition of 50 μl of D2O and 50 μl of 0.75% TSP. ^1^H-MR spectra of the extracted and media samples were acquired at 25°C using a pulse-acquired MR sequence with water suppression (1D NOESY presat sequence) in a broad-band-inverse NMR probe on a Bruker 500 MHz NMR system.

### Sulforhodamine B (SRB) assay

Cells were plated in appropriate tissue culture vessels, and allowed to adhere overnight prior to treatment. At the end of incubation, media was removed and cells were fixed with 10% trichloroacetic acid (TCA) for 30 min. TCA was washed with water, wells were allowed to air dry, and then an excess of 0.4% (w/v) SRB in 1% acetic acid was used to stain fixed cells for >10 min. Excess SRB was washed off with 1% acetic acid solution. Bound SRB was resuspended in a suitable volume of 10 mM Tris, and absorbance of solution measured at 570 nm. For proliferation assays, cells were plated on “day −1” in triplicate in 12 well plates, and cultured in maintenance DMEM overnight, after which “day 0” plates fixed with TCA. For galactose conditions, glucose media was replaced with glucose-free DMEM, supplemented with 4.5 g/L galactose, 10% FCS, penicillin, streptomycin and L-glutamine, and 1 mM pyruvate. Cells were then incubated for desired time points in either normoxia or hypoxia, followed by SRB assay.

### Experimental design, data analysis, and statistical procedures

Data are presented as mean ± standard deviation (S.D) or standard error (S.E.M). Statistical analysis was performed using an unpaired two-tailed *t-*test in GraphPad Prism version 7.02 for Windows (La Jolla, California, USA) or otherwise indicated in figure legends. Statistical significance was determined using *P* < 0.05.

## Author contributions

TB and JMS designed and performed experiments, analyzed data, and contributed to writing the manuscript. LWT, CE, and SES performed experiments and analyzed data. Y-LC performed metabolomics analysis and analyzed data. MT performed electron microscopy. LAM performed MiNA analysis. BG performed bioinformatics analysis. PHM provided RCC10 pVHL mutant cells. GS and SV designed experiments. MA provided the concept for the study, designed experiments, analyzed data, wrote the manuscript, and acquired funding. All authors reviewed the manuscript.

### Conflict of interest statement

The authors declare that the research was conducted in the absence of any commercial or financial relationships that could be construed as a potential conflict of interest.
