# Corrigendum: VHL-Mediated Regulation of CHCHD4 and Mitochondrial Function

**DOI:** 10.3389/fonc.2021.740273

**Published:** 2021-09-23

**Authors:** Thomas Briston, Jenna M. Stephen, Luke W. Thomas, Cinzia Esposito, Yuen-Li Chung, Saiful E. Syafruddin, Mark Turmaine, Lucas A. Maddalena, Basma Greef, Gyorgy Szabadkai, Patrick H. Maxwell, Sakari Vanharanta, Margaret Ashcroft

**Affiliations:** ^1^ Division of Medicine, Centre for Cell Signalling and Molecular Genetics, University College London, London, United Kingdom; ^2^ Department of Medicine, University of Cambridge, Cambridge, United Kingdom; ^3^ Cancer Research UK Cancer Imaging Centre, Institute of Cancer Research London, London, United Kingdom; ^4^ Medical Research Council Cancer Unit, Hutchison/MRC Research Centre, University of Cambridge, Cambridge, United Kingdom; ^5^ Division of Biosciences, Department of Cell and Developmental Biology, University College London, London, United Kingdom; ^6^ The Francis Crick Institute, London, United Kingdom; ^7^ Department of Biomedical Sciences, University of Padua, Padua, Italy; ^8^ Cambridge Institute for Medical Research, University of Cambridge, Cambridge, United Kingdom

**Keywords:** Hippel-Lindau protein (pVHL), hypoxia inducible factor, mitochondria, bioenergetics, metabolism, CHCHD4, respiratory chain

In the original article, there was a mistake in **Figure 4A** as published. The figure text was mislabeled, and a lane-line was missing. The corrected **Figure 4** appears below.

**Figure 4:**
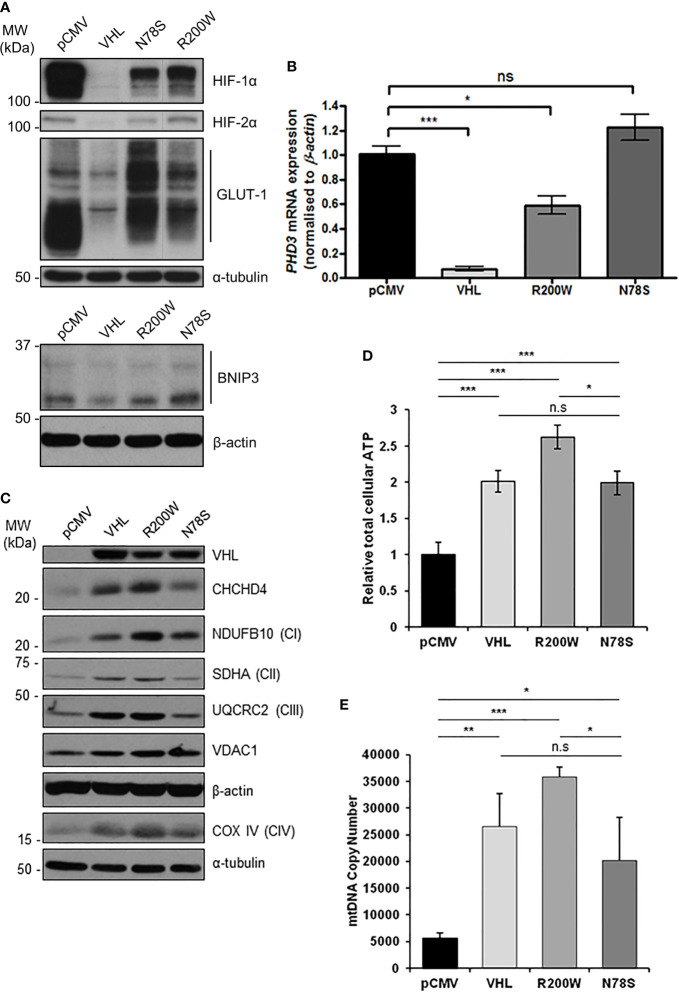
pVHL mutants differentially regulate mitochondrial protein expression, mtDNA copy number and ATP levels. **(A)** Western blots show HIF-1α, HIF-2α, GLUT-1, and BNIP3 protein levels in RCC10 cells expressing empty vector (pCMV), wild type pVHL (VHL), or pVHL mutants (R200W or N78S). α-tubulin and β-actin were used as load controls. **(B)** Relative expression of PHD3 mRNA in RCC10 cells described in **(A)**, measured using RT-qPCR. Data were analyzed using the comparative Ct method. Data are presented as mean ± S.E.M. n = 3 (n.s. p > 0.05, *p < 0.05, and ***p < 0.001). **(C)** Western blots show expression of mitochondrial proteins CHCHD4 and VDAC1, and respiratory chain subunits NDUFB10 (CI), SDHA (CII), UQCRC2 (CIII), COX IV (CIV) in RCC10 cells described in **(A)**. pVHL expression was assessed as a control for re-expression, and β-actin and α-tubulin were used as load controls. **(D)** Graph shows total cellular ATP content in RCC10 cells expressing wild type pVHL (VHL) or pVHL mutants (R200W or N78S), normalized to cell number (n = 4). **(E)** Graph shows mtDNA copy number in RCC10 cells expressing pVHL variants, calculated using the ratio of expression of mitochondrial ND1 gene to the single copy nuclear gene, *β*2M to by RT-qPCR. Data in **(D, E)** are presented as mean ± S.D. n = 6 (n.s. p > 0.05, *p < 0.05, **p < 0.01, and ***p < 0.001).

The authors apologize for this error and state that this does not change the scientific conclusions of the article in any way. The original article has been updated.

## Publisher’s Note

All claims expressed in this article are solely those of the authors and do not necessarily represent those of their affiliated organizations, or those of the publisher, the editors and the reviewers. Any product that may be evaluated in this article, or claim that may be made by its manufacturer, is not guaranteed or endorsed by the publisher.

