# Supplementary material for: VHL-Mediated Regulation of CHCHD4 and Mitochondrial Function
Source: Front Oncol. 2018 Oct 4;8:388. doi: 10.3389/fonc.2018.00388 (PMC6180203; doi:10.3389/fonc.2018.00388)
Supplement: Supplementary file 1 [file Data_Sheet_1.pdf]

Supplementary Material

VHL-mediated regulation of CHCHD4 and mitochondrial function.

Thomas Briston[1¶§], Jenna M. Stephen[2§], Luke W. Thomas[2], Cinzia Esposito[2], Yuen-Li Chung[3], Saiful E. Syafruddin[4], Mark Turmaine[5], Lucas A. Maddalena[2], Basma Greef[2], Gyorgy Szabadkai[5], Patrick Maxwell[6], Sakari Vanharanta[4] and Margaret Ashcroft[2*].

*** Author for correspondence**

University of Cambridge, Cambridge, CB2 0AH, United Kingdom.

Tel: +44 (0)122 376 2024

Email: m.ashcroft@medschl.cam.ac.uk

Complex	Protein	Genetic Origin	Change in expression	Reference
I	NADH-ubiquinone oxidoreductase 24kDa (NDUFA9)	Nucleus	No change (cells)<br>Increased (tissue)	19
III	Ubiquinol cytochrome c reductase core protein 1 (UQCRC1)	Nucleus	Increased	19
III	Ubiquinol cytochrome c reductase core protein 2 (UQCRC2)	Nucleus	Increased	19
IV	Cytochrome c oxidase subunit 2 (mtCO-2)	Mitochondria	Increased	18
IV	Cytochrome c oxidase subunit 4 (COX-IV)	Nucleus	Increased	18
V	ATP synthase FO subunit 6 (ATP6)	Mitochondria	Increased	18
V	ATP synthase, mitochondrial F_1 complex alpha (ATP5A)	Nucleus	No change	18
V	ATP synthase, mitochondrial F_1 complex beta (ATP5B)	Nucleus	No change	18

Supplementary Table 1: pVHL regulates mitochondrial respiratory chain protein expression in renal carcinoma cells. Table shows mitochondrial protein, genetic origin, the change observed in response to pVHL re-expression, and reference.

Gene	Forward Primer	Reverse Primer
β-actin	CCCAGAGCAAGAGAGG	GTCCAGACGCAGGATG
COX-IV	GAGCAATTTCCACCTCTGT	CAGGAGGCCTTCTCCTTCTC
Cytochrome b	TATCCGCCATCCCATACATT	GGTGATTCCTAGGGGGTTGT
PHD3	GATGCTGAAGAAAGGGC	CTGGCAAAGAGAGTATCTG
HIF-2α/EPAS	AAGCCTTGGAGGGTTTCATTG	TGCTGATGTTTTCTGACAGAAA
mtCO-2	TTCATGATCACGCCCTCATA	TAAAGGATGCGTAGGGATGG
ND6	AGGTAGGATTGGTGCTGTGG	CCAATAGGATCCTCCCGAAT
SDHA	GGACAACTGGAGGTGGCATT	TTTTCTAGCTCGACCACGGC
TFAM	CCGAGGTGGTTTTTCATCTGT	ACGCTGGGCAATTCTTCTAA
PGC-1α	TGATGACAGCGAAGATGA	AGAAGAACAAGAAGGAGACA
PGC-1β	ACACTGACTACGATTCCAA	TCTGAGGTATTGAGGTATTCC
NRF-1	GCAGCCGCTCTGAGAACTTCA	TCGTAAGAGGTGTCCTCGGG
NRF-2	CACACTTCTAGTCGCGGGAG	CAAAGGATCAGTCCCACTC

Supplementary Table 2: Primer sequences. Table shows the sequences of primers for used for PCR, and were obtained from Sigma Aldrich.

Construct	Oligo
**Control shRNA**<br><br>*Renilla* Ctrl	TGCTGTTGACAGTGAGCGCAGGAATTATAATGCTTATCTATAGTGAAGCCACAG<br>ATGTATAGATAAGCATTATAATTCCTATGCCTACTGCCTCGGA
**HIF-2α shRNA (1)**<br><br>*shEPAS1_4*	TGCTGTTGACAGTGAGCGACCAGGTGAAAGTCTACAACAATAGTGAAGCCACAG<br>ATGTATTGTTGTAGACTTTCACCTGGCTGCCTACTGCCTCGGA
**HIF-2α shRNA (2)**<br><br>*shEPAS1*_9	TGCTGTTGACAGTGAGCGCCCAGCAGATGGACAACTTGTATAGTGAAGCCACAG<br>ATGTATACAAGTTGTCCATCTGCTGGTTGCCTACTGCCTCGGA

Supplementary Table 3: Table shows the shRNA oligonucleotide sequences used for targeting *HIF2A* (*EPAS1*), and were obtained from Sigma Aldrich.

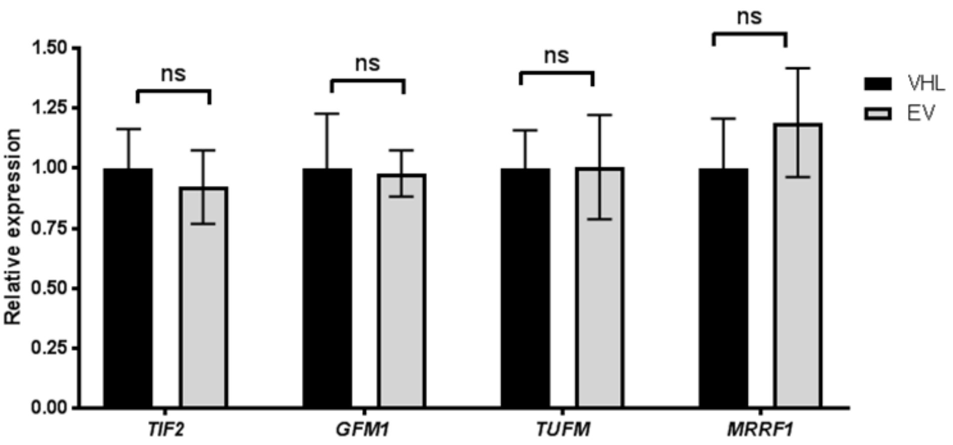

Supplementary Figure 1: pVHL re-expression does not alter the transcript levels of major regulators of mitochondrial translation. Relative expression of *TIF2*, *GFM1*, *TUFM* and *MRRF1* analyzed using RT-qPCR. Data were analyzed using the comparative Ct method. Data are presented as mean ± S.E.M. n=4 (n.s. $p>0.05$).

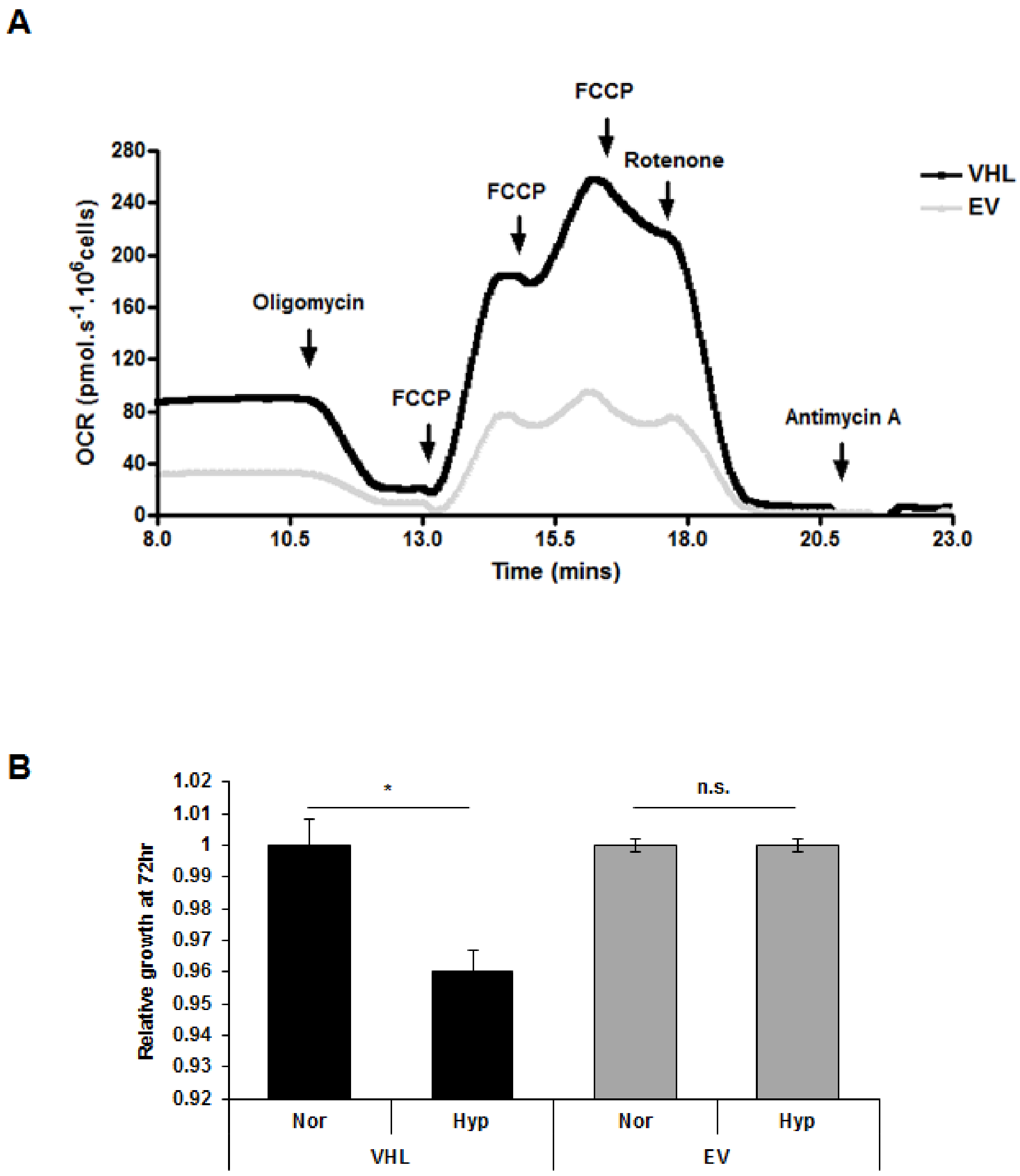

Supplementary Figure 2: Effects of pVHL status on basal OCR and growth in hypoxia. (A) Representative oxygen flux analysis in 786O-VHL (VHL) and 786O-EV (EV) cells using the Oroboros Oxygraph 2K. Leak respiration was determined using oligomycin (2 µg/ml) and titration of FCCP (0.5 µM) to determine maximum respiratory capacity. Non-mitochondrial respiration was determined through the combined administration of rotenone (0.5 µM) and antimycin A (2.5 µM) to inhibit complexes I and III respectively. Data are corrected for cell number. (B) Graph shows relative growth of cells described in A, after exposure to normoxia (Nor) or hypoxia (1% O_2, Hyp) for 72 hours (72 hr). Total cell protein was measured using SRB. Hypoxia values were normalized to normoxia values. Data are presented as mean ±S.D, n=2 independent experiments (n.s. $p>0.05$, * $p<0.05$).

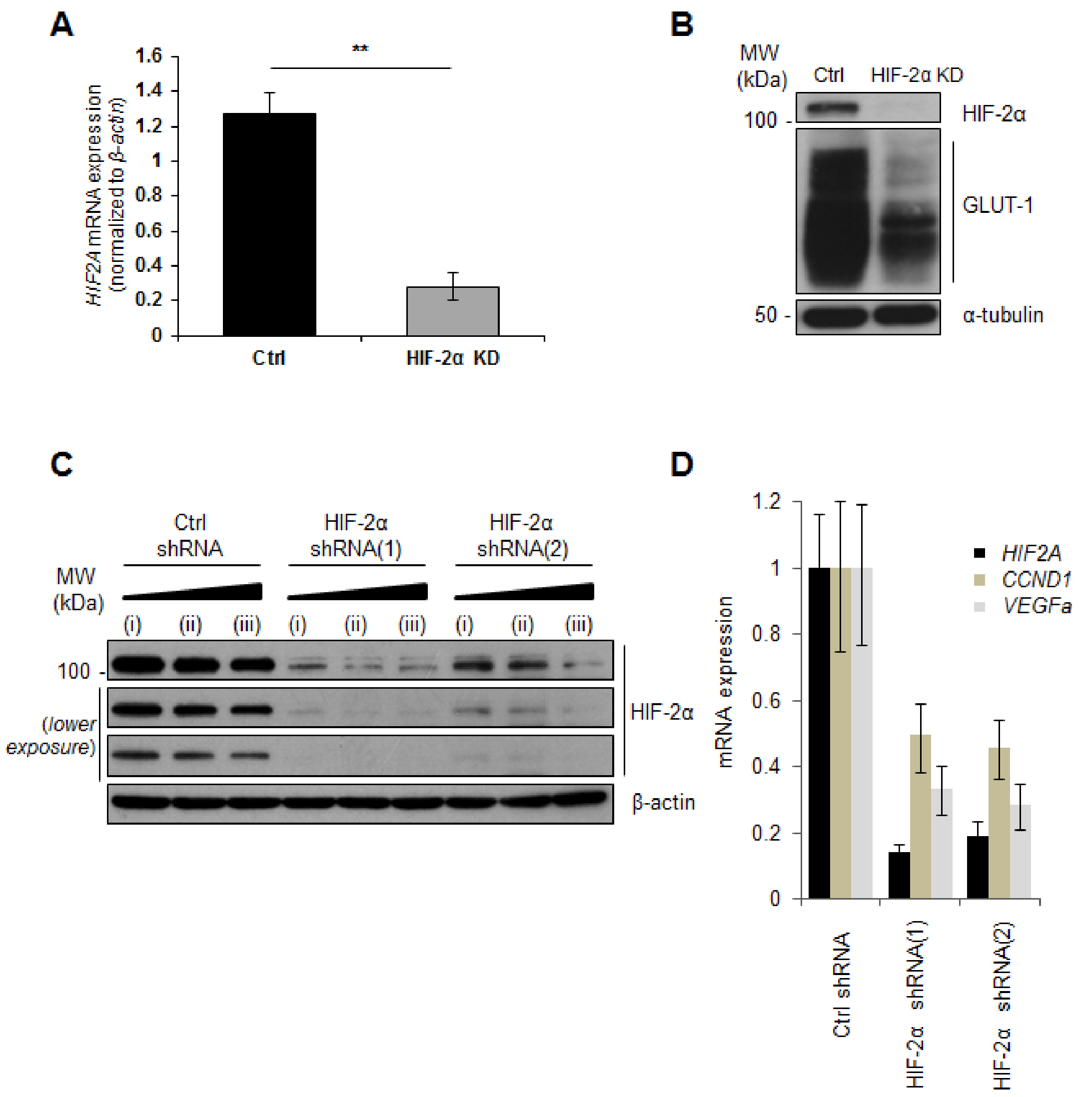

Supplementary Figure 3: HIF-2α knockdown in 786O cells. (A) Relative expression of *HIF-2A* mRNA (normalized to *β-actin*) in cells in 786O-EV cells transiently expressing a non-silencing control siRNA (Ctrl) or HIF-2α siRNA (HIF-2α KD), measured using RT-qPCR. Data analyzed using the comparative Ct method. Data are presented as mean ± S.E.M. n=4 (** $p<0.01$). (B) Western blots show HIF-2α and GLUT1 protein levels in cells described in A. α-tubulin was used as a load control. (C) Western blots show HIF-2α protein levels in independent 786O cell pools stably transduced with increasing amounts ((i) 100 µL, (ii) 200 µL and (iii) 300 µL) of shRNA lentivirus expressing control shRNA (Ctrl shRNA) and two independent HIF-2α shRNAs (1) and (2). β-actin was used a load control. (D) Expression of *HIF2A* mRNA and its downstream targets, *CCND1* and *VEGFA* in independent 786O cell pools (ii) described in C, as measured by qRT-PCR. Data are presented as mean ± 95% C.I. for three technical replicates.

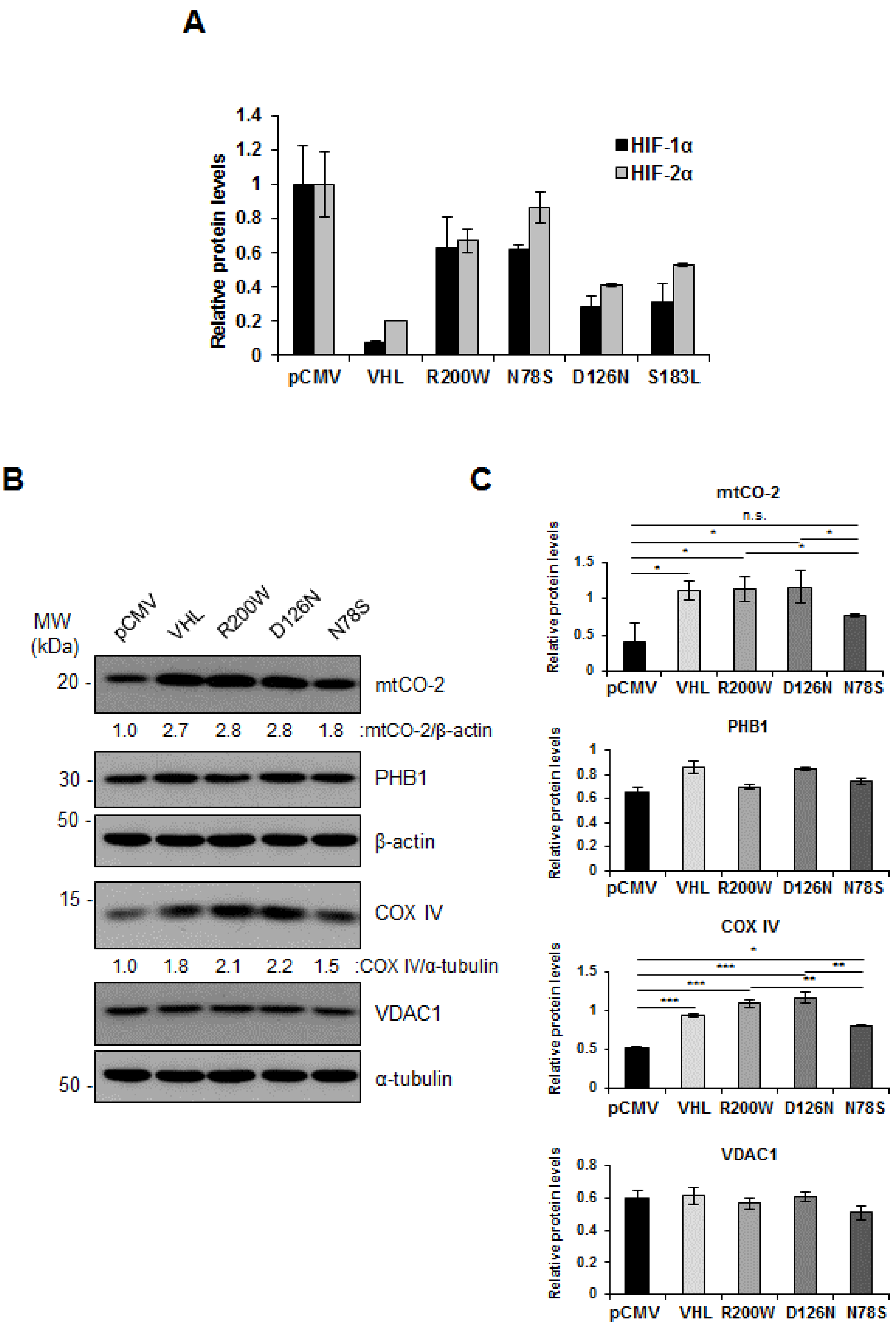
A
Relative protein levels
1.4
1.2
1
0.8
0.6
0.4
0.2
0
HIF-1α
HIF-2α
pCMV VHL R200W N78S D126N S183L
B
MW (kDa)
pCMV VHL R200W D126N N78S
20 -
mtCO-2
1.0 2.7 2.8 2.8 1.8 :mtCO-2/β-actin
30 -
PHB1
50 -
β-actin
15 -
COX IV
1.0 1.8 2.1 2.2 1.5 :COX IV/α-tubulin
VDAC1
50 -
α-tubulin
C
mtCO-2
n.s.
Relative protein levels
pCMV VHL R200W D126N N78S
PHB1
Relative protein levels
pCMV VHL R200W D126N N78S
COX IV
Relative protein levels
pCMV VHL R200W D126N N78S
VDAC1
Relative protein levels
pCMV VHL R200W D126N N78S

D

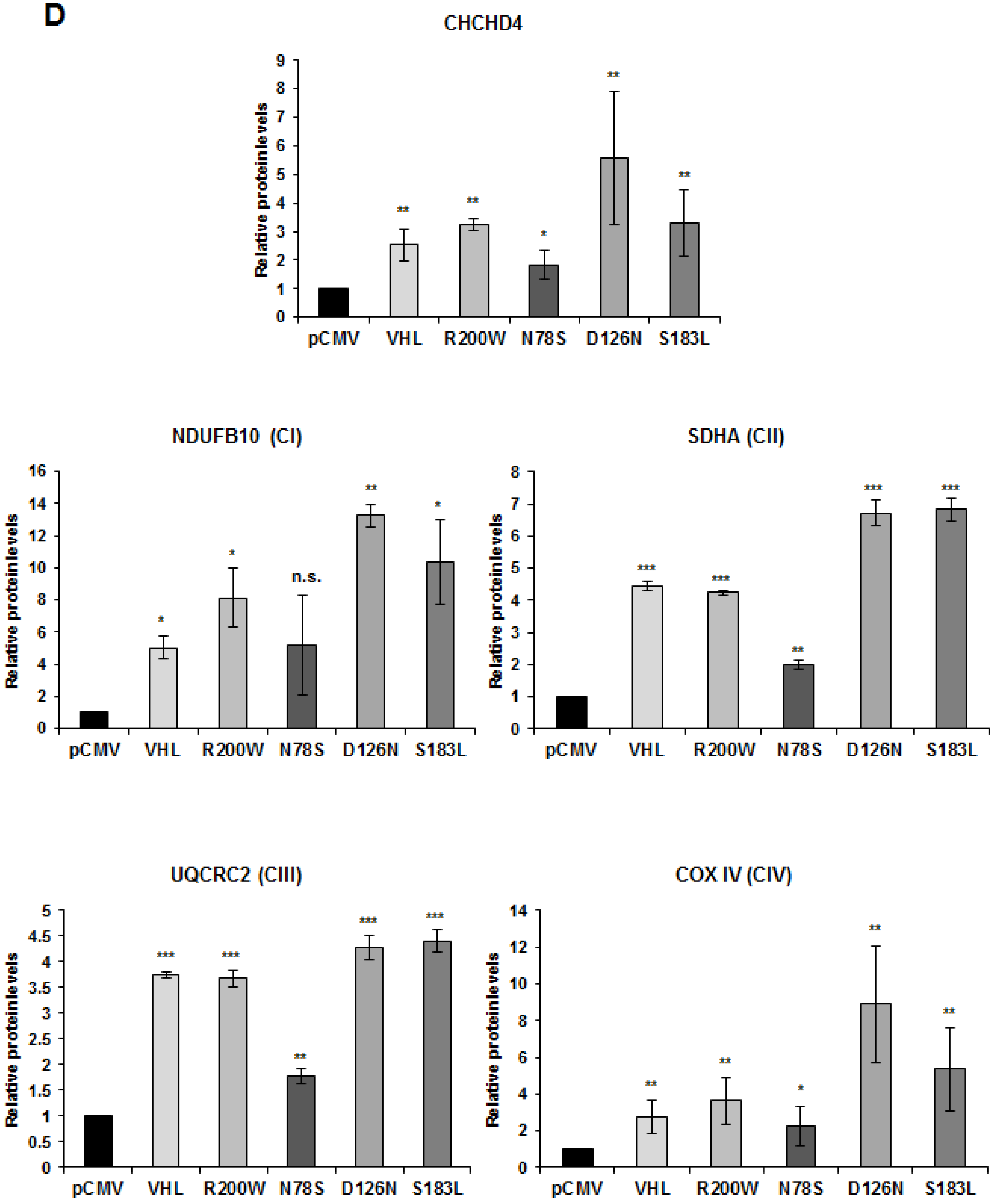
CHCHD4
Relative protein levels
pCMV
VHL
R200W
N78S
D126N
S183L
NDUFB10 (CI)
n.s.
SDHA (CII)
UQCRC2 (CIII)
COX IV (CIV)

Supplementary Figure 4: pVHL mutants differentially regulate mitochondrial protein expression. (A) Graphs show densitometric analysis of western blots of RCC10 cells expressing empty vector (pCMV), wild type pVHL (VHL) or pVHL mutants (R200W, N78S, D126N and S183L). Relative protein levels (arbitrary units) are shown for HIF-1α and HIF-2α proteins. (B) Western blot analysis of cells described in A. Mitochondrial proteins mtCO-2, COX IV, PHB1 and VDAC1 are shown. β-actin and α-tubulin was used as a load control. Densitometric analysis of Western blots show fold change in mtCO-2 and COX IV proteins normalized to the load and relative to the pCMV control. n=3. (C-D) Graphs show relative levels of mtCO-2, COX IV, PHB1 and VDAC1 (C) and CHCHD4, NDUFB10, SDHA, UQCRC2 and COX IV (D) proteins from densitometric analysis of western blots from experiments described in A-B. Values were normalized to the load. Data are presented as mean ± S.D. (n.s. $p>0.05$, * $p<0.05$, ** $p<0.01$ and *** $p<0.001$).

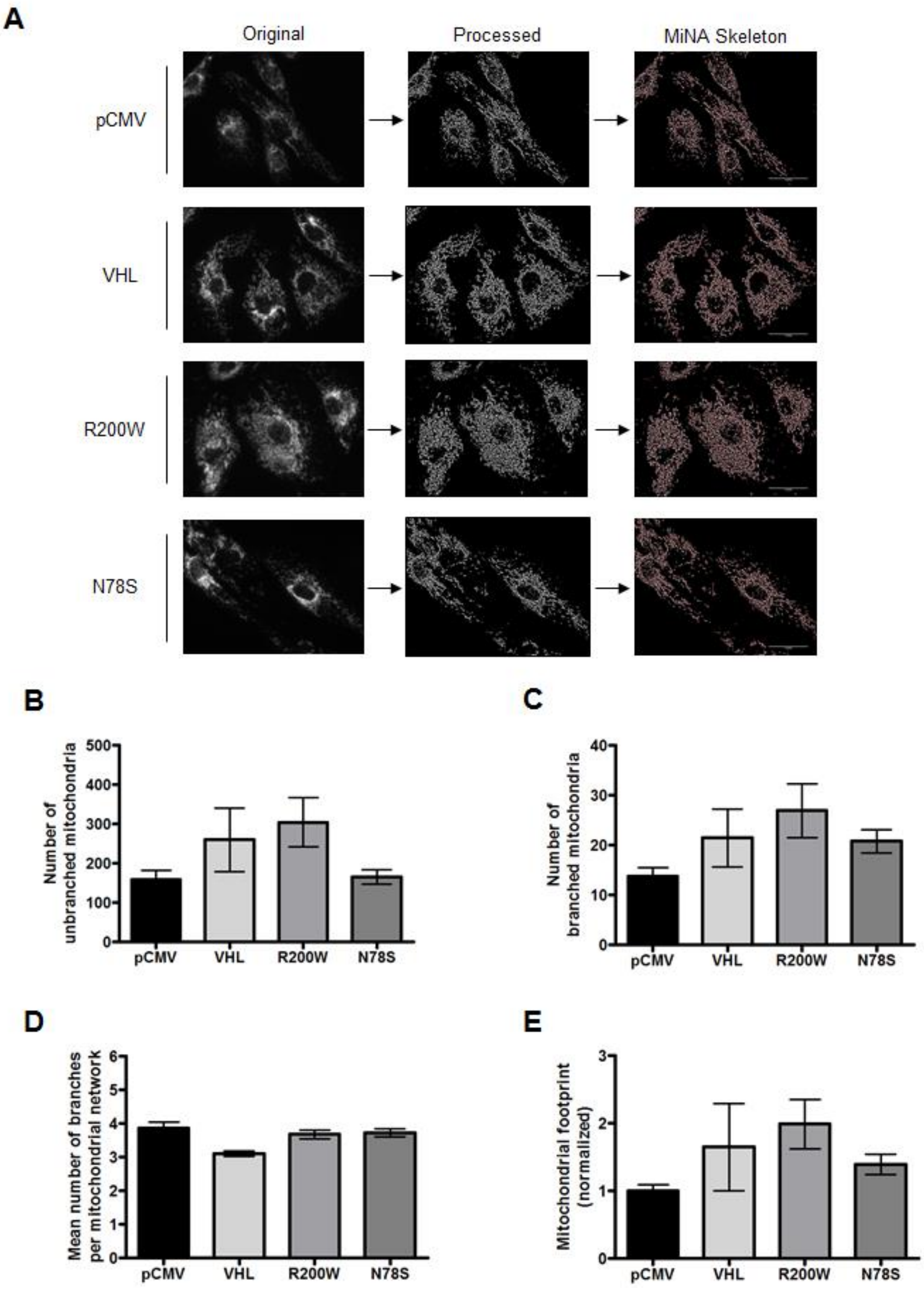
A
Original
Processed
MiNA Skeleton
pCMV
VHL
R200W
N78S
B
Number of unbranched mitochondria
500
400
300
200
100
0
pCMV
VHL
R200W
N78S
C
Number of branched mitochondria
40
30
20
10
0
pCMV
VHL
R200W
N78S
D
Mean number of branches per mitochondrial network
6
5
4
3
2
1
0
pCMV
VHL
R200W
N78S
E
Mitochondrial footprint (normalized)
3
2
1
0
pCMV
VHL
R200W
N78S

F

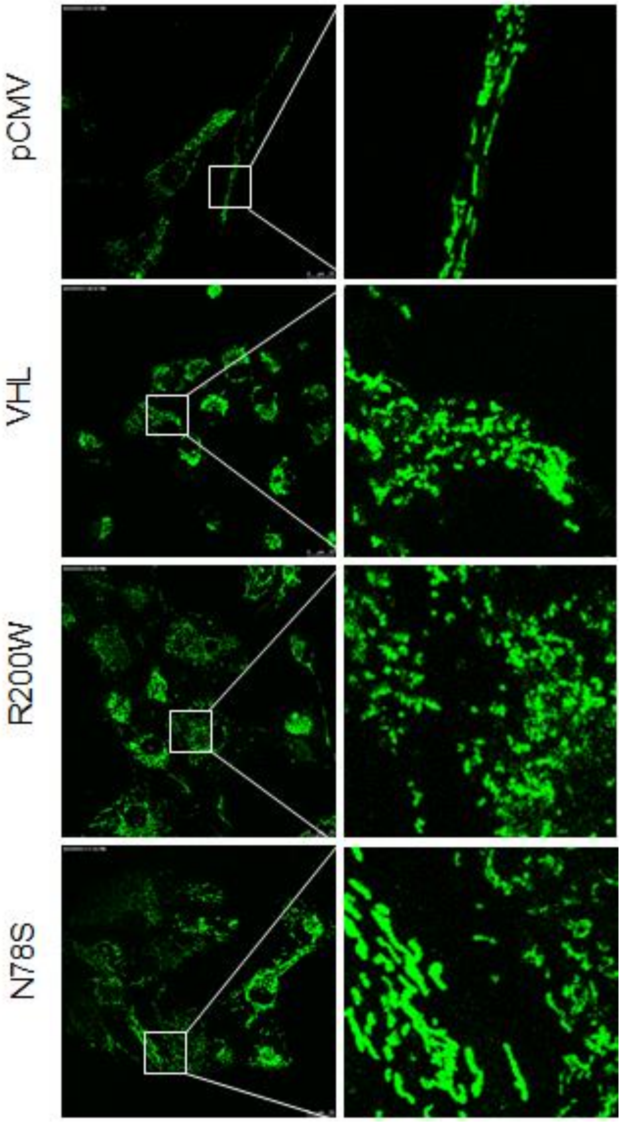

Supplementary Figure 5: pVHL mutants differentially regulate mitochondrial morphology and the mitochondrial network. (A) Confocal microscope images of RCC10 cells empty vector (pCMV), wild type pVHL (VHL) or pVHL mutants (R200W or N78S), stained with ATPB as described in Figure 6A. Images were analyzed using MiNA [39] and data represented graphically in Figure 6B-E. (B-E) Images of cells described in A stained with MitoTraker red. Graphs show analysis of mitochondria using MiNA [39]. Graphs show number of mitochondria that are unbranched (B) and branched (C), mean number of branched mitochondria per network (D) and the mitochondrial footprint normalized to pCMV control (E) for each cell line as indicated. n=15 (pCMV), n= 4 (VHL), n=18 (R200W), n=16 (N78S). Data are presented as mean ± S.E.M and analyzed using a one-way ANOVA with Tukey's post-hoc comparison. (F) Confocal microscope images of live RCC10 cells described in A stained with MitoView Green (100 nM).

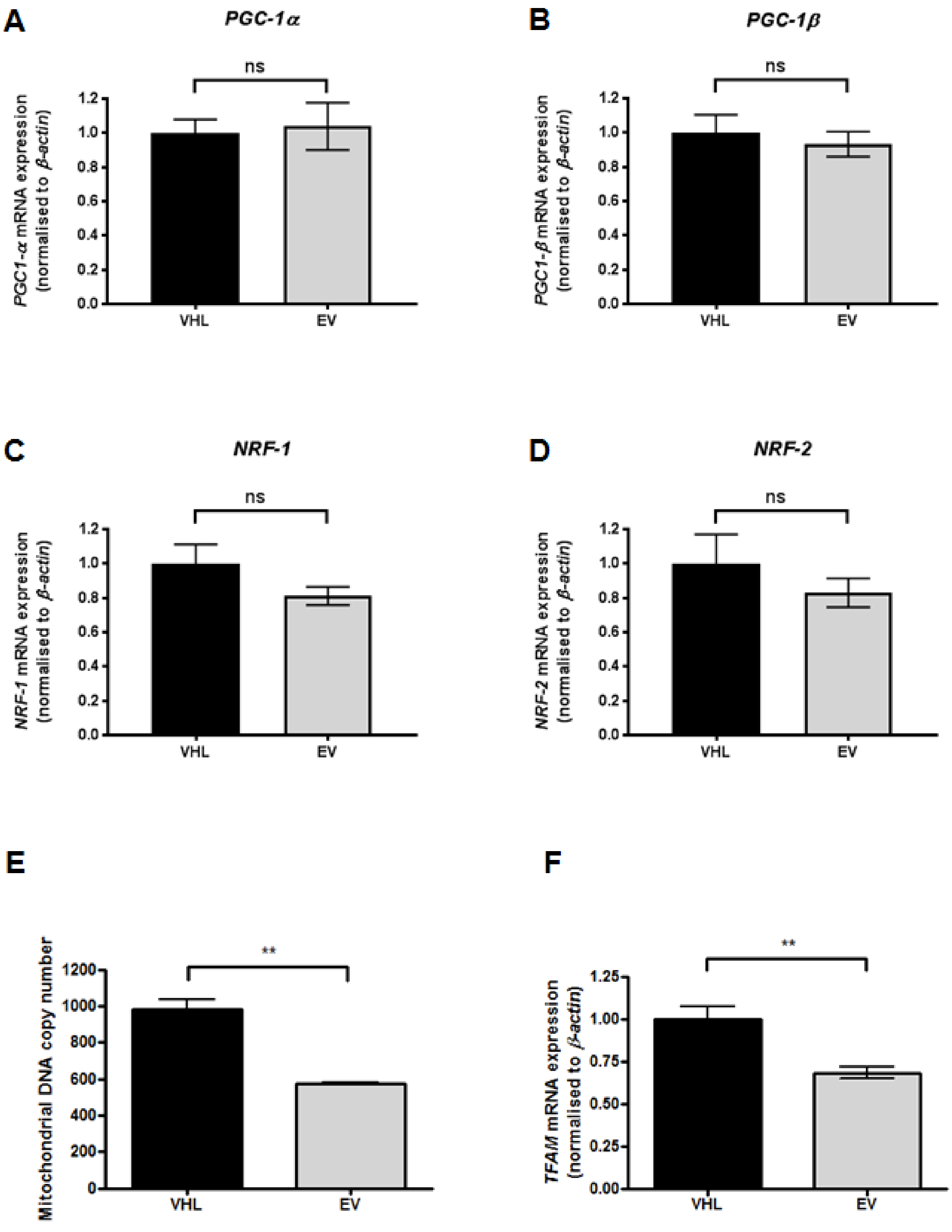
A
PGC-1α
ns
PGC1-α mRNA expression (normalised to β-actin)
1.2
1.0
0.8
0.6
0.4
0.2
0.0
VHL
EV
B
PGC-1β
ns
PGC1-β mRNA expression (normalised to β-actin)
VHL
EV
C
NRF-1
ns
NRF-1 mRNA expression (normalised to β-actin)
VHL
EV
D
NRF-2
ns
NRF-2 mRNA expression (normalised to β-actin)
VHL
EV
E
**
Mitochondrial DNA copy number
1200
1000
800
600
400
200
0
VHL
EV
F
**
TFAM mRNA expression (normalised to β-actin)
1.25
1.00
0.75
0.50
0.25
0.00
VHL
EV

G

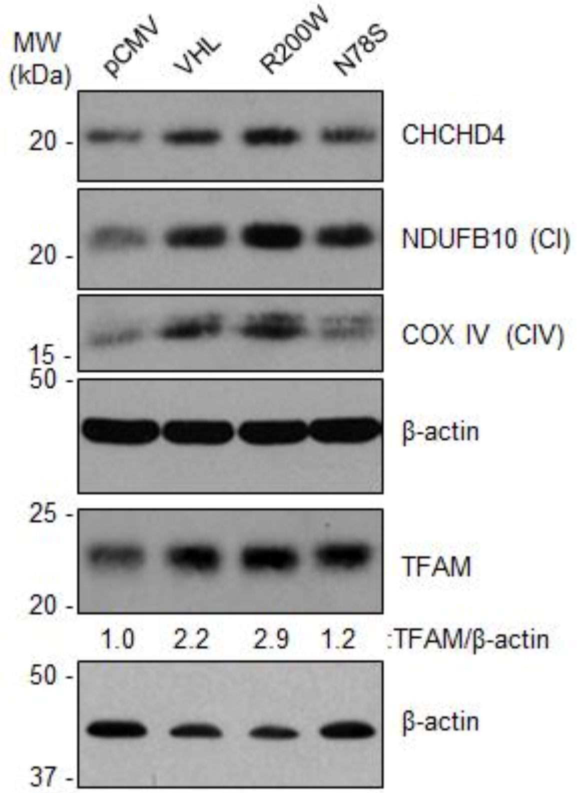

Supplementary Figure 6: pVHL re-expression increases mtDNA copy number without affecting mRNA expression of major regulators of mitochondrial biogenesis. (A-D) Relative expression of *PGC-1α*, *PGC-1β*, *NRF-1* and *NRF-2* mRNA in 786O-VHL (VHL) and 786O-EV (EV) cells, measured using RT-qPCR. Data were analyzed using the comparative Ct method. (E) Mitochondrial DNA copy number in 786O-VHL (VHL) and 786O-EV (EV) cells, calculated using the ratio of expression of mitochondrial *ND1* gene to the single copy nuclear gene, *β2M* to analyzed by RT-qPCR (n=5). (F) Relative expression of *TFAM* mRNA in 7860-VHL and 786O-EV cells, measured using RT-qPCR. Data were analyzed using the comparative Ct method. Data are presented as mean ± S.E.M. n=8 (n.s. p>0.05 and ** p<0.01). (G) Western blots show CHCHD4, NDUFB10, COX IV and TFAM protein levels in RCC10 cells empty vector (pCMV), wild type pVHL (VHL) or pVHL mutants (R200W or N78S). β-actin was used as a load control.
